# MAP3K13-232aa encoded by circMAP3K13 enhances cisplatin-induced pyroptosis by directly binding to IKKα in gastric adenocarcinoma

**DOI:** 10.1038/s41419-025-07991-5

**Published:** 2025-09-01

**Authors:** Kaining Du, Xiaojing Zhang, Ying Qin, Huizhen Ma, Chuhan Bing, Shiqi Deng, Yang Chen, Jiequan Qin, Shanshan Chang, Siyu Xiao, Lehua Peng, Xiaoya Xie, Xianling Feng, Xianli Fu, Yanjie Wei, Xinmin Fan, Hassan Ashktorab, Duane Smoot, Zhe Jin, Yin Peng

**Affiliations:** 1 Guangdong Provincial Key Laboratory of Genome Stability and Disease Prevention, Department of Pathology, Shenzhen University Health Science Center, Shenzhen, Guangdong PR China; 2 Guangdong Provincial Key Laboratory of Regional Immunity and Diseases, Department of Pathology, Shenzhen University Medical School, Shenzhen University, Shenzhen, Guangdong PR China; 3 Department of Pathology, Guangdong Province Key Laboratory of Molecular Oncologic Pathology, Shenzhen University Medical School, Shenzhen University, Shenzhen, Guangdong PR China; 4 Department of Gastrointestinal Surgery, The First Affiliated Hospital of Shenzhen University, Shenzhen Second People’s Hospital, Shenzhen, Guangdong PR China; 5 Department of Pathology, Shenzhen University General Hospital, Shenzhen, Guangdong PR China; 6 Center for High Performance Computing, Shenzhen Institutes of Advanced Technology, Shenzhen, Guangdong PR China; 7 Department of Medicine and Cancer Center, Howard University, College of Medicine, Washington, DC USA; 8 Department of Medicine, Meharry Medical Center, Nashville, TN USA

**Keywords:** Tumour biomarkers, Non-coding RNAs

## Abstract

Gastric cancer (GC) is one of the most common and lethal malignancies in developing countries, with particularly high prevalence in China. Circular RNAs (circRNAs) have garnered increasing attention for their roles in disease pathogenesis. While circRNAs can be translated, there have been few investigations into the biological functions of “translatable circRNAs” in the initiation and progression of gastric adenocarcinoma. In this study, we identified a circRNA, circMAP3K13, which inhibits the proliferation and migration of GC cells. CircMAP3K13 was found to encode a previously unreported 26 kDa protein, designated MAP3K13-232aa. Mechanistically, MAP3K13-232aa binds directly to the kinase domain of IKKα and enhances its activity, thereby promoting NF-κB signaling. This activation leads to upregulation of NLRP3 and increased cisplatin-induced pyroptosis in GC cells. Moreover, MAP3K13-232aa enhances pyroptosis and reduces tumorigenicity and metastasis in vivo. Taken together, both circMAP3K13 and its encoded protein MAP3K13-232aa represent potential therapeutic targets in GC.

## Introduction

Gastric cancer (GC) is one of the most common malignant tumors and ranks as the fourth leading cause of cancer-related deaths worldwide [1]. Despite considerable efforts to improve GC treatment, the prognosis for patients diagnosed at an advanced stage remains poor [2]. Efforts to elucidate the mechanisms of gastric carcinogenesis and identify potential diagnostic and therapeutic biomarkers are, therefore, crucial to reduce the high mortality rate associated with GC.

Circular RNAs (circRNAs) have recently attracted attention for their roles in disease pathogenesis and their potential use as biomarkers, owing to their relatively high stability. First discovered in viroids [3], circRNAs were later identified in mammals and other species [4]. Advances in high-throughput sequencing technologies have led to the discovery of numerous circRNAs involved in the pathogenesis of various human diseases [5, 6]. Further research revealed that circRNAs can function as microRNA sponges, protein decoys, transcriptional regulators, and even encode peptides or proteins that contribute to normal cellular physiology [7–11]. Due to their covalently closed loop structure and translational potential [12], circRNAs are more stable than linear mRNAs and hold promise as therapeutic agents in future cancer treatments. Given their unique features, it is important to investigate the role of circRNAs in cancer and their underlying mechanisms of action.

A growing body of research has shown that nuclear factor (NF)-κB participates in inflammation and immune responses, and regulates cell proliferation, apoptosis, stress responses, and tumorigenesis [13]. IκB kinase (IKK)α is a component of the IKK complex that regulates the NF-κB pathway. Upon phosphorylation by IKKα, IκB is degraded, allowing NF-κB to translocate into the nucleus and regulate the transcription of target gene [14, 15]. Thus, activation of IKKα is essential for NF-κB pathway activation. NF-κB is also a known transcriptional activator of NLRP3, a component of the inflammasome, with NF-κB-binding sites identified in the *NLRP3* promoter region [16]. The NLRP3 inflammasome acts as a sensor for pyroptosis, a form of inflammatory programmed cell death [17]. The adaptor protein ASC mediates the interaction between NLRP3 and pro-caspase 1, leading to caspase 1 activation. Activated caspase 1 cleaves gasdermin D (GSDMD) and pro-IL-1β and pro-IL-18. The N-terminal fragment of cleaved GSDMD forms membrane pores, leading to the release of cellular contents, IL-1β, and IL-18 [18], causing cell rupture and an amplified inflammatory response [19].

During cancer development, pyroptosis influences tumor cell proliferation, invasion, metastasis, and modulation of the tumor immune microenvironment [20]. In GC, GSDMD suppresses the expression of cyclin A2 and CDK2 by inhibiting ERK1/2, STAT3, and PI3K/AKT pathways, thereby arresting the cell cycle in S phase. By contrast, silencing GSDMD enables enables GC cells to progress from S to G2 phase [21]. Moreover, the NLRP3–ASC–Caspase-1–IL-1β signaling cascade contributes to antitumor immunity [22, 23], highlighting the role of pyroptosis in regulating both cancer cell survival and the immune microenvironment.

Although NF-κB is a critical upstream activator of pyroptosis, the underlying mechanisms of NF-κB-mediated pyroptosis in GC are still unknown. In this study, we identified circRNAs that were differentially expressed in at least four GC cell lines when compared with two normal gastric mucosal epithelial cell lines. Among them, circMAP3K13, derived from the *MAP3K13* gene, was found to encode a novel protein, MAP3K13-232aa. We demonstrate that MAP3K13-232aa binds directly to the kinase domain of IKKα and enhances its kinase activity. Ultimately, MAP3K13-232aa activates the NF-κB signaling pathway and increases cisplatin-induced pyroptotic activity in GC cells.

## Methods

### Tissue samples

All GC and paired normal tissue samples were collected from patients at Shenzhen Second People’s Hospital, China. Informed, written consent was obtained from each patient, and the study was approved by the Ethics Committee of Shenzhen University School of Medicine. Pathological information for all patients is provided in the supplementary files.

### Cell culture

GC cell lines (AGS, MKN-28, NCI-N87, HGC-27, MKN-45), immortalized normal human gastric epithelial cells (HFE-145, GES-1), and HEK293T cells were all obtained from the Department of Pathology, Shenzhen University. AGS, MKN-28, NCI-N87, HGC-27, MKN-45, and GES-1 cells were cultured in RPMI-1640 medium (Hyclone, Logan, UT, USA) supplemented with 10% fetal bovine serum (FBS; Gibco, Grand Island, NY, USA). HFE-145 and HEK293T cells were cultured in DMEM (Hyclone) supplemented with 10% FBS. All cells were maintained at 37 °C in a humidified atmosphere containing 5% CO_2_. All cell lines were tested for mycoplasma regularly and tested for STR profiling. To induce NF-κB pathway activity, cells were treated with 20 ng/mL TNF-α for 5 min.

### RNA sequencing (RNA-seq)

Six cell lines (AGS, MKN-28, SGC-7901, BGC-823, GES-1, and HFE-145) were subjected to RNA seq and ribosome profiling sequencing (Ribo-seq). Total RNA was extracted using TRIzol reagent (Invitrogen, Cat. No. 251808). Libraries were constructed using the NEBNext Multiplex Small RNA Library Prep Set for Illumina (Cat. No. E7300S, E7300L). RNA-seq and Ribo-seq were performed by Gene Denovo Biotechnology (Guangzhou, China) using the Illumina HiSeq™ 2500 platform. Twenty-mers from both ends of the unmapped reads were extracted and aligned to the reference genome using TopHat2 to identify unique anchor positions within splice sites. Anchor reads that were aligned in a reversed (head-to-tail) orientation were considered indicative of circRNA splicing and were analyzed using the “find_circ” tool to identify circRNAs. CircRNA quantification was performed by scaling back-spliced junction reads to Reads Per Million mapped reads (RPM).

### Plasmids and construction of stable transfected MKN-45 cell lines

Full-length circMAP3K13-3×FLAG and a version containing a mutated open reading frame (ORF) start codon circMAP3K13-3xFLAG were chemically synthesized and cloned into the pLC5-ciR vector (Geneseed, Guangzhou, China) using the EcoRI and BamHI restriction enzymes (Thermo Fisher Scientific, USA). A version of the circMAP3K13 overexpression plasmid lacking the downstream flanking sequence was used as a negative control. The CMV-MAP3K13-232aa linear overexpression vector served as a positive control. HEK293T cells were transfected with both control plasmids, a circMAP3K13 overexpression plasmid, a linear MAP3K13-232aa overexpression plasmid, and helper packaging plasmids to produce viral supernatant. A total of 1 mL of viral supernatant was added to each well of a 6-well plate to infect MKN-45 cells. After 4–6 h of infection, the medium was replaced with fresh culture medium, and cells were incubated for an additional 24 h. Puromycin was then added at a final concentration of 2 μg/mL to select for stable transfectants. Stable MKN-45 cell lines were established after 2–3 weeks of selection.

### Fluorescence in situ hybridization (FISH)

Fluorescent probes were obtained from RiboBio (Guangzhou, China). FISH was performed using a Fluorescent In Situ Hybridization Kit (RiboBio), following the manufacturer’s instructions.

### Reverse-transcription PCR and quantitative PCR

Total RNA was extracted as described, and RNA was reverse-transcribed into cDNA using the GoScript Reverse Transcription Mix (Promega, Cat. Nos. A2800 and A6002). Quantitative real-time PCR was performed using the GoTaq qPCR Master Mix (Cat. Nos. A6001 and A6002, Promega). U6 and 18S were used as internal reference genes. Primer sequences are listed in the supplementary files.

### RNase R treatment assay

RNase R (Geneseed, R0301; 1 U/μg) was added to total RNA (4 μg) and incubated at 37 °C for 10 min. Digestion products were purified using the RNeasy MinElute Cleanup Kit (Qiagen). The purified RNA was then used for reverse-transcription (RT) PCR and quantitative PCR.

### Dual-luciferase reporter assay

Predicted full-length and truncated internal ribosome entry site (IRES) sequences were cloned into the Luc2 empty vector to generate IRES reporter plasmids. These constructs were transfected into HEK293T cells. After 48 h, luciferase activity was measured using the Dual-Glo® Luciferase Assay System (Promega), following the manufacturer’s instructions. The ratio of firefly to Renilla luciferase activity was compared between experimental groups to assess relative IRES activity.

### Western blotting

Cells were lysed in 2× Laemmli sample buffer (Bio-Rad) containing protease inhibitors (Roche, Basel, Switzerland) and incubated on ice for 10 min. Lysates were then adjusted to equal concentrations, and proteins were denatured at 95 °C for 10 min. Equal volumes of protein lysates were loaded onto 10% SDS-PAGE gels, separated by electrophoresis, and then transferred to PVDF membranes (Millipore, Bedford, MA, USA). Membranes were blocked with 5% non-fat milk for 1 h at room temperature and then incubated with primary antibodies overnight at 4 °C. The next day, membranes were incubated with secondary antibodies for 1 h at room temperature. Immunoreactive signals were detected using enhanced chemiluminescence reagent. The western blot signals were quantified using ImageJ.

### Antibodies

An anti-FLAG antibody (F1804) was obtained from Sigma-Aldrich (St. Louis, MO, USA). Antibodies against Bcl-2 (#2875), Bcl-XL (#2764), XIAP (#14334), c-IAP2 (#3120), cyclin D1 (#55506S), and IKKα (#2682), and the Pyroptosis Antibody Sampler Kit (#43811) were purchased from Cell Signaling Technology (Danvers, MA, USA). The anti–c-IAP1 antibody against c-IAP1 (sc-271419) was from Santa Cruz Biotechnology (Dallas, TX, USA). Antibodies against MAP3K13 and MAP3K13-232aa (OTI8A4) were obtained from Origene (Rockville, MD, USA). The NF-κB Signaling Pathway Antibody Panel (ab228529) was purchased from Abcam (Cambridge, UK). The anti-NLRP3 antibody (T55651) was from Abmart (Shanghai, China). Antibodies against GST-tag (66001-2-Ig) and His-tag (66005-1-Ig) were provided by Proteintech Group (Rosemont, IL, USA). The anti-GAPDH antibody (AC002) was purchased from ABclonal (Wuhan, China). All primary antibodies were used at a dilution of 1:1000, and secondary antibodies were diluted by 1:2000.

### Immunoprecipitation (IP)

Cells were lysed in IP lysis buffer containing protease inhibitors (Roche), 48 h after transfection. Lysates were incubated on ice for 10 minto ensure complete protein release, followed by centrifugation at 12,000 rpm for 10 min to collect the supernatant. For FLAG-tagged proteins, lysates were incubated overnight at 4 °C with anti-FLAG magnetic beads (Sigma-Aldrich). For other targets, lysates were incubated with the appropriate primary antibody overnight at 4 °C, followed by incubation with protein A/G magnetic beads (Thermo Fisher Scientific, USA) for 1 h at room temperature. After washing, bound proteins were eluted and analyzed by western blotting.

### GST pull down assay

Plasmids encoding GST-tagged or His-tagged proteins were constructed using the pGEX-4T1-GST and pET28A-His vectors, respectively. Recombinant plasmids and corresponding empty vectors (EV) were transformed into BL21 chemically competent cells (KT Life Technology, Shenzhen). Expressed proteins by BL21 cells were purified using GSTSep Glutathione Agarose Resin or HisSep Ni-NTA Agarose Resin (Yeasen Biotechnology, Shanghai), following the manufacturers’ protocols. GST pull-down assays were performed to identify the IKKα domain that directly interacts with MAP3K13-232aa. To reduce non-specific binding to the GST beads (Yeasen Biotechnology), 100 mM NaCl was added to both the lysis solution and wash buffers.

### Kinase activity assays

IKKα was enriched by IP. The kinase activity of IKKα and MAP3K13-232aa was assessed by ADP-Glo Kinase Assay (Promega, USA). IκBα substrate was obtained from LMAI Bio (Shanghai, China), and the generic MBP substrate was purchased from MCE, respectively.

### LDH-release assays

AGS cells were transfected with either an empty vector (EV) or an overexpression plasmid. After 48 h, lipopolysaccharide (LPS; 2 μg/mL) was added for 4 h, followed by nigericin (20 μM) for 1 h to induce pyroptosis. Lactate dehydrogenase (LDH) levels in the supernatant were measured using an LDH Cytotoxicity Assay Kit (Beyotime, Shanghai, China), according to the manufacturer’s instructions.

### Wound healing assay

Transfected cells were seeded into 6-well plates and cultured until approximately 80% confluence. Once fully confluent, 3–5 linear wounds of equal width were made using a 200-μL pipette tip. After washing with PBS to remove debris, cells were incubated at 37 °C in 5% CO₂. Images of the wound area were captured at 0 h, 24 h, 48 h, and 72 h to assess cell migration.

### Transwell invasion assay

Transwell chambers pre-coated with Matrigel matrix were used to evaluate the invasive capacity of AGS or HGC-27 cells that had been transfected with plasmids or siRNAs for 48 h. Serum-free medium containing 1–5 × 10^5^/mL cells was added to the upper Transwell chamber, while medium supplemented with 10% FBS was added into the lower chamber. After 48 h of incubation, invasive cells on the lower membrane surface were fixed with 4% paraformaldehyde for 30 min and stained with 0.1% crystal violet for 30 min. Non-invasive cells were removed, and migrated cells were counted under a microscope (Nikon, Tokyo, Japan).

### EDU cell proliferation assay

Cell proliferation was assessed using the Cell-Light EdU Apollo567 In Vitro Kit (RiboBio, Guangzhou), following the manufacturer’s instructions. The cell proliferation rate of transfected GC cells was evaluated by laser-scanning confocal microscopy (ZEISS LSM880, Oberkochen, Germany).

### Colony-formation assays

A total of 1000 cells were seeded into 6-well plates containing complete growth medium and incubated at 37 °C with 5% CO_2._ The medium was replaced every 3 days. After 2–3 weeks, cell colonies were fixed with 4% paraformaldehyde for 30 min and stained with 0.1% crystal violet for 30 min. Colonies were then counted, and the rates of colony formation rates were calculated.

### In vivo carcinogenesis and metastasis assay

Stably transfected MKN-45 cells were used for in vivo experiments. Six-week-old female BALB/c nude mice were randomly divided into three groups (n = 6 per group) and subcutaneously injected with 1 × 10^6^ stably transfected MKN-45 cells resuspended in 100 μL PBS to establish xenograft tumor models. The tumor volume of each group of mice was measured every other day. On day 23 after injection, mice were sacrificed, and the tumors were excised and weighed. For the tumor metastasis model, 1 × 10^6^ stably transfected MKN-45 cells were injected into the tail vein of 6-week-old female BALB/c nude mice. After 7 weeks, the mice were sacrificed, and lung tissues were collected for hematoxylin and eosin (H&E) staining to evaluate the numbers of metastatic foci in each mouse. All animal experiments were approved by the Animal Care Committee of Shenzhen University Health Science Center.

### Statistical analyses

All statistical analyses were performed using GraphPad Prism software (version 8.0.2; USA). Chi-square tests and two-tailed Student’s t-tests were used to determine statistical significance. Data are presented as the means ± standard deviation (SD) from at least three independent experiments. A *P* < 0.05 was considered statistically significant difference.

The sequences of primers and siRNAs (Supplementary Table 1), as well as patient pathological information (Supplementary Table 2), are provided in the supplementary materials.

## Results

### circRNA screening identifies circMAP3K13 differentially expressed in GC

**Fig. 1:**
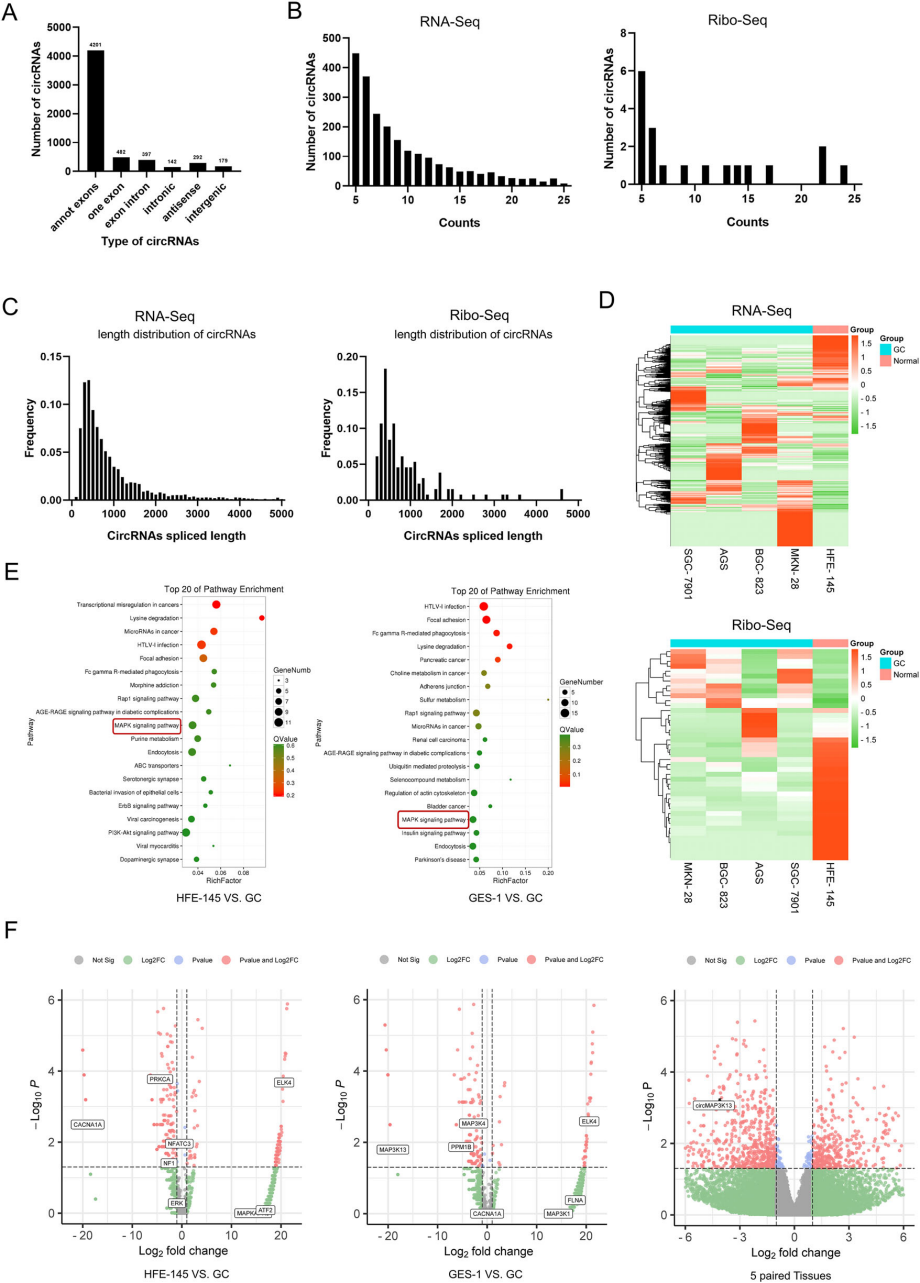
RNA-seq and Ribo-seq screening of differentially expressed circRNAs in GC. To construct circRNA expression profiles, RNA-seq and Ribo-seq were performed on two normal human gastric mucosal epithelial cell lines (GES-1 and HFE-145) and four human gastric cancer (GC) cell lines (AGS, MKN-28, SGC-7901, and BGC-823) using the Illumina HiSeq™ 2500 platform. **A** Classification of circRNAs detected by RNA-seq, grouped by circRNA type. **B** Distribution of circRNAs counts, as detected by RNA-seq and Ribo-seq. **C** Length distribution of circRNAs, as identified by RNA-seq and Ribo-seq. **D** Heatmap showing gene expression profiles from RNA-seq and Ribo-seq analyses across GC and normal cell lines. **E** KEGG pathway enrichment analysis showing the top 20 significantly enriched pathways in differentially expressed genes between normal (GES-1 and HFE-145) and GC cells. **F** Volcano plots of differentially expressed genes in GES-1 vs. GC and HFE-145 vs. GC comparisons, highlighting MAPK pathway-associated genes (left and middle). RNA-seq data from five pairs of GC and adjacent normal tissues. circMAP3K13 (right, black arrow) was detected in five paired GC tissues and adjacent normal tissues by RNA-seq.

To identify differentially expressed circRNAs with translation potential in GC, we performed RNA-seq and Ribo-seq on four human GC cell lines (AGS, MKN-28, SGC-7901, BGC-823) and two normal human gastric mucosal epithelial cell lines (GES-1, HFE-145). The total reads were mapped to the reference genome using TopHat2 and Bowtie2. We obtained 5,693 circRNAs from the RNA-seq results and 132 circRNAs from Ribo-seq across the six cell lines (Fig. 1A, B). Most identified circRNAs were <1000 nucleotides, with a notable enrichment between 300 and 400 nucleotides in length both in RNA-seq and Ribo-seq datasets (Fig. 1C). This size distribution is consistent with previous reports, as translatable circRNAs are typically shorter [24].

Gene cluster analysis revealed distinct patterns of differential gene expression in GC cell lines compared with normal gastric epithelial cells (Fig. 1D). KEGG enrichment analysis of the differentially expressed genes in the RNA-seq dataset showed significant enrichment of the MAPK signaling pathway (indicated by a high Q-value; Fig. 1E), suggesting its involvement in GC initiation and progression. Among the differentially expressed genes within this pathway, eight—*MAP3K13*, *PPM1B*, *MAP3K4*, *ELK4*, *CACNA1A*, *PRCKA*, *NF1*, and *NFATC3*—showed significant fold changes in GC cells (Fig. 1F).

In a previous study, we performed RNA-seq on five pairs of GC tissues and matched adjacent normal tissues and identified dysregulated circRNAs in GC [25]. One of the significantly downregulated circRNAs was hsa_circ_11430 (http://reprod.njmu.edu.cn/cgibin/circrnadb/detail_info.php?circ_id=hsa_circ_11430), derived from the MAP3K13 gene (Fig. 1F, Supplementary Fig. 1A). Additionally, MAP3K13 has been implicated in tumorigenesis [26] (Supplementary Fig. 1B). Based on these findings, we selected hsa_circ_11430—hereinafter referred to as circMAP3K13—for further investigation into its biological role in GC.

### CircMAP3K13 is expressed at low levels in GC cells and tissues

**Fig. 2:**
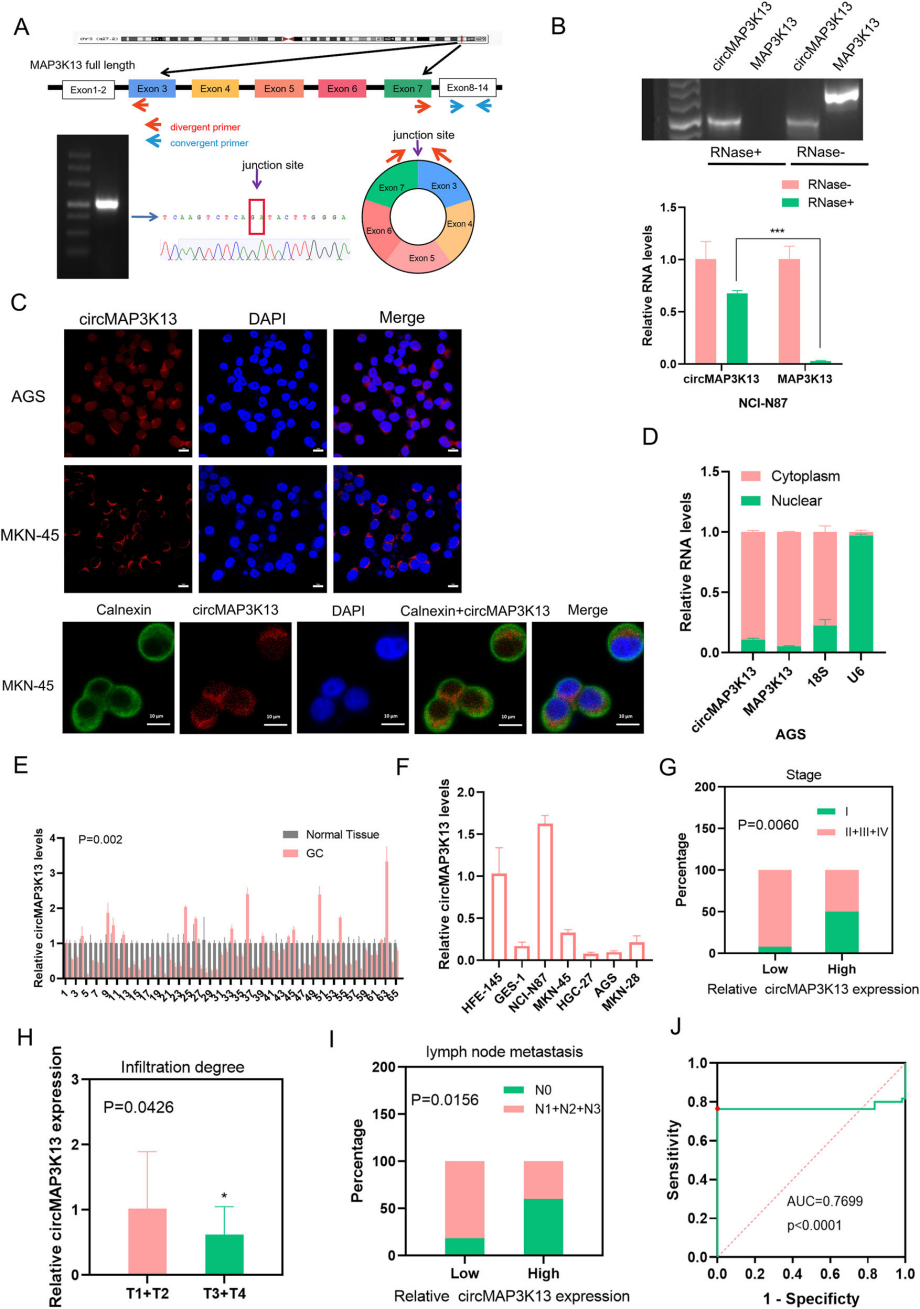
Expression of circMAP3K13 in GC. **A** Schematic of the *MAP3K13* gene (upper), consisting of 14 exons; circMAP3K13 is encoded by exons 3–7. Divergent and convergent primers were designed to detect circMAP3K13 and linear MAP3K13, respectively. DNA agarose gel detecting circMAP3K13 in AGS cells (lower, left) Sanger sequencing results (lower, middle), and the junction site of circMAP3K13 between exons 3 and 7 (lower, right). **B** Total RNA was treated with RNase R, and the digested RNA was analyzed by qPCR to assess the expression of circMAP3K13 and MAP3K13. DNA agarose gel displays the qPCR products. **C** FISH was performed to determine the localization of circMAP3K13 (red) in AGS and MKN-45 cells. Co-staining with the endoplasmic reticulum marker Calnexin (green) was carried out in MKN-45 cells. Nuclei are counterstained with DAPI (blue). **D** qPCR analysis of circMAP3K13 in nuclear and cytoplasmic fractions of AGS cells. 18S and U6 served as cytoplasmic and nuclear controls, respectively. **E** Bar chart showing qPCR results for circMAP3K13 expression in 65 paired GC (pink) and adjacent non-tumor tissues (gray); *P* = 0.002. **F** CircMAP3K13 expression in GC cell lines. **G** CircMAP3K13 expression in GC patients. The percentage of patients with low expression in stage I gastric cancer was higher than that of patients with low expression in stages Ⅱ, Ⅲ and Ⅳ. The Chi-square test was used to analyze the data. **H** circMAP3K13 expression in tumors with different infiltration degrees (n = 48 patients). The higher the expression of circMAP3K13, the deeper the invasion depth of the tumor. T1: invasion into the mucosal or muscular layer; T2: invasion into the subserosal layer or outer serosa; T3: penetration of the gastric wall with serosal involvement; T4: invasion beyond the serosa or into adjacent structures. **I** Association between circMAP3K13 expression and lymph node metastasis. N0: no metastasis; N1: 1–2 nodes; N2: 3–6 nodes; N3: 7 or more nodes. Chi-square test was used. **J** Receiver operating characteristic curve for circMAP3K13 expression in GC versus adjacent tissues. The reference line (red dotted), ROC curve (green), and cutoff point (red dot) are shown. If not otherwise specified, data were analyzed using two-tailed Student’s t-test. *P < 0.05; **P < 0.01; ***P < 0.001.

CircMAP3K13 is formed from exons 3–7 of the *MAP3K13* gene and is composed of 803 nucleotides. A divergent primer was designed to amplify the back-splice junction site of circMAP3K13 in AGS cells, and the junction site sequence was confirmed by Sanger sequencing (Fig. 2A). The presence of circMAP3K13 in AGS and MKN-45 cells was also confirmed by PCR using divergent primers on cDNA, but not genomic DNA (Supplementary Fig. 1C). To assess its stability, total RNA from NCI-N87 cells was treated with RNase R. Quantitative PCR analysis of the digested product revealed that circMAP3K13, unlike its linear counterpart, was resistant to RNase R digestion (Fig. 2B), consistent with the known stability of circRNAs [27].

To determine the subcellular localization of circMAP3K13, FISH was performed in AGS and MKN-45 cells. The results showed that circMAP3K13 is predominantly localized in the cytoplasm. Notably, circMAP3K13 co-localized with the endoplasmic reticulum (ER) marker Calnexin in MKN45 cells (Fig. 2C), suggesting potential for translation into a novel protein, as the ER is a central site for protein synthesis.

FISH was also performed under knockdown and overexpression conditions of circMAP3K13-232aa to confirm the specificity of the FISH probe (Supplementary Fig. 1D). Quantitative PCR analysis further demonstrated that circMAP3K13 was enriched in the cytoplasm of AGS cells compared to the nucleus (Fig. 2D). These results indicate that circMAP3K13 is primarily cytoplasmic and associates with the ER in GC cells.

To assess the expression levels of circMAP3K13 in human tissues, we performed quantitative PCR on 65 paired samples of GC and adjacent normal tissues. CircMAP3K13 expression was significantly lower in GC tissues than that in matched normal adjacent tissues (*P* = 0.002) (Fig. 2E). We also evaluated circMAP3K13 expression across a panel of gastric cell lines, including two normal gastric mucosal cell lines (HFE145 and GES-1) and five GC cell lines (AGS, HGC-27, MKN-28, MKN-45, and NCI-N87). Overall, we found that circMAP3K13 expression was consistently lower in GC cell lines compared with that in HFE-145 (Fig. 2F).

### circMAP3K13 expression negatively correlates with tumor progression

To evaluate the clinical significance of circMAP3K13 in GC, we analyzed its association with clinicopathological features in a cohort of 48 patients. We considered tumor-node-metastasis stage, depth of infiltration, and lymph node metastasis. CircMAP3K13 expression was negatively associated with cancer stage, showing higher expression in early-stage cases and lower expression in later stages (II–IV) (Fig. 2G). Similarly, circMAP3K13 expression was significantly higher in tumors with lower infiltration grades (T1 and T2) compared to those with more advanced infiltration (T3 and T4) (Fig. 2H). In addition, patients with fewer lymph node metastases exhibited higher circMAP3K13 expression, indicating an inverse relationship between circMAP3K13 levels and lymph node involvement (Fig. 2I). These findings suggest that low circMAP3K13 expression is associated with advanced tumor progression in GC.

To assess its diagnostic potential, we generated a receiver operating characteristic curve based on circMAP3K13 expression levels in GC tissues and paired adjacent normal tissues. The resulting area under the curve was 0.7699 (*P* < 0.0001), indicating good discriminatory power for GC diagnosis (Fig. 2J). A cutoff value of 0.913 was identified as a potential diagnostic threshold. Together, these results imply that circMAP3K13 expression negatively correlates with GC progression and may serve as a diagnostic biomarker for GC diagnosis.

### CircMAP3K13 encodes a new protein MAP3K13-232aa

**Fig. 3:**
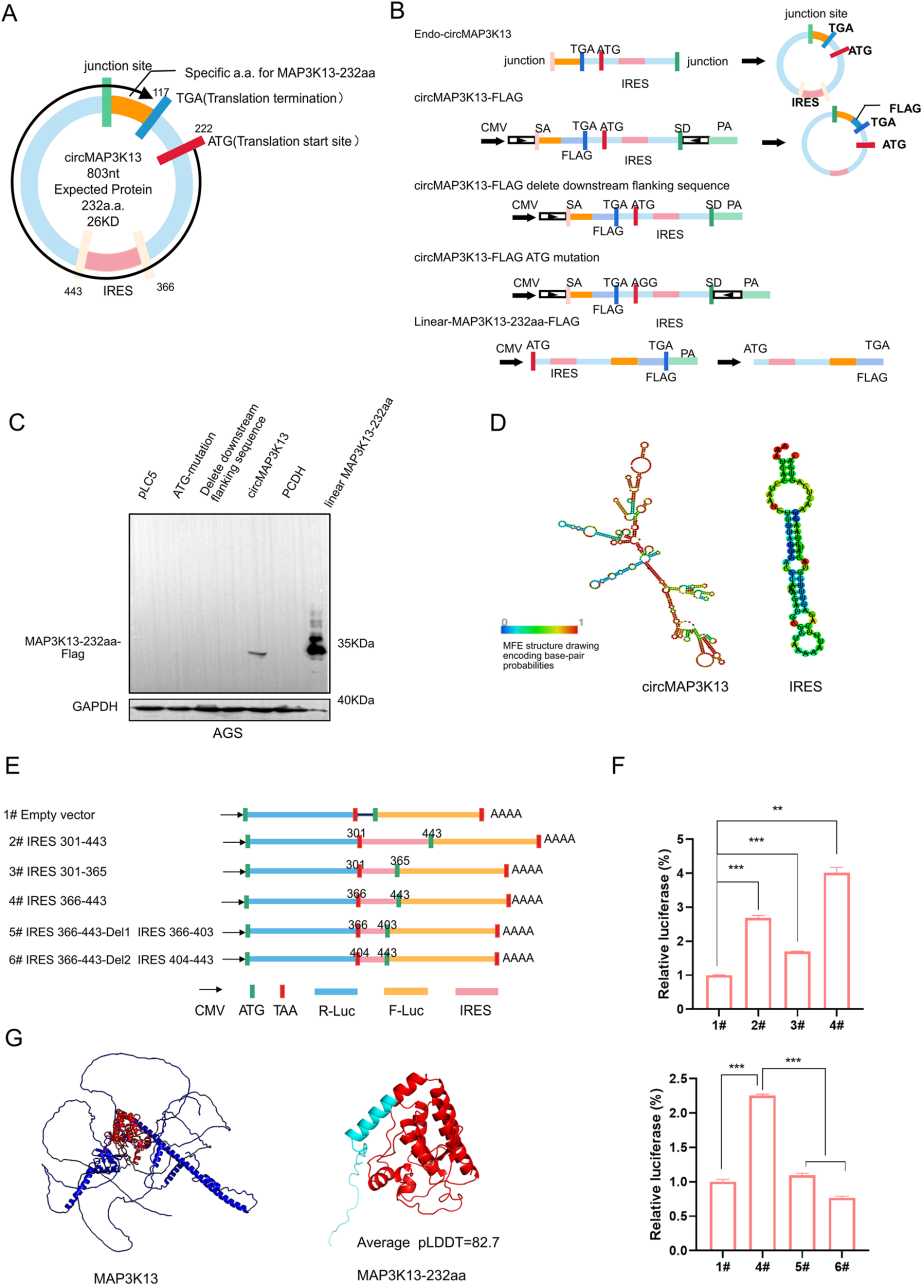
circMAP3K13 harbors an active IRES sequence, and can be translated into a novel protein. **A** Schematic of circMAP3K13, which consists of 803 nucleotides and is predicted to encode a novel protein of approximately 26 kDa. **B** Schematic representation of plasmid constructs. The circMAP3K13-FLAG plasmid overexpresses circMAP3K13 with a 3×FLAG tag. The downstream flanking sequence deletion mutant lacks the downstream complementary sequence required for circularization. The ATG-mutation construct disrupts the start codon, altering the open reading frame. The linear-MAP3K13-232aa-FLAG plasmid expresses the same ORF of circMAP3K13 in a linear form. **C** Western blot analysis of circMAP3K13-FLAG and linear-MAP3K13-232aa-FLAG. **D** RNAfold software prediction of the secondary structure of circMAP3K13 and a putative internal ribosome entry site (IRES). **E** Schematic of five recombinant plasmids generated to assess IRES activity by dual-luciferase reporter assay. **F** Relative luciferase activity of plasmids 1–6. **G** AlphaFold3–based 3D structure prediction of MAP3K13-232aa. The MAP3K13-232aa sequence is highlighted in red within the full MAP3K13 protein (left) and the specific MAP3K13-232aa peptide is shown in cyan (right). Unless otherwise specified, statistical analysis was performed using a two-tailed Student’s t-test. **P < 0.01; ***P < 0.001.

The circMAP3K13 sequence contains a putative ORF predicted to encode a 232–amino acid (aa) protein, referred to hereafter as MAP3K13-232aa, with an approximate molecular weight of 26 kDa, as predicted by the circRNAdb database (Fig. 3A). To further confirm that circMAP3K13 indeed encodes this novel protein MAP3K13-232aa, we constructed a circMAP3K13-FLAG-tagged circMAP3K13 overexpression plasmid by inserting a 3×FLAG sequence upstream of the “TGA” stop codon. Two negative-control plasmids were generated: one with the downstream flanking sequence deleted, and another with a mutated ORF start codon (referred to as ATG-mutant circMAP3K13). In parallel, we constructed a plasmid expressing MAP3K13-232aa in a linear expression plasmid encoding MAP3K13-232aa (Fig. 3B). Only the plasmid with wild-type circMAP3K13 plasmid produced a detectable protein, with a molecular size matching that of the linear MAP3K13-232aa (Fig. 3C), supporting that the putative ORF of circMAP3K13 encodes a novel protein.

As circRNAs lack a 5’ cap structure, translation of circMAP3K13 requires an IRES. Using IRESite (http://www.iresite.org/) and RNAfold (http://rna.tbi.univie.ac.at/cgi-bin/RNAWebSuite/RNAfold.cgi) software, we identified a potential IRES element within nucleotides 366–443. A dual-luciferase reporter assay revealed that only the wild-type IRES366–443, and not the truncated version, significantly induced significant luciferase expression, indicating that IRES366–443 initiates 5′-cap-independent translation (Fig. 3D–F).

To further characterize the structure of the novel protein, we predicted the 3D structure of MAP3K13-232aa using AlphaFold 3 (Fig. 3G). The structure revealed seven α-helices, covering much of the core kinase domain of MAP3K13, suggesting that it may have kinase activity.

**Fig. 4:**
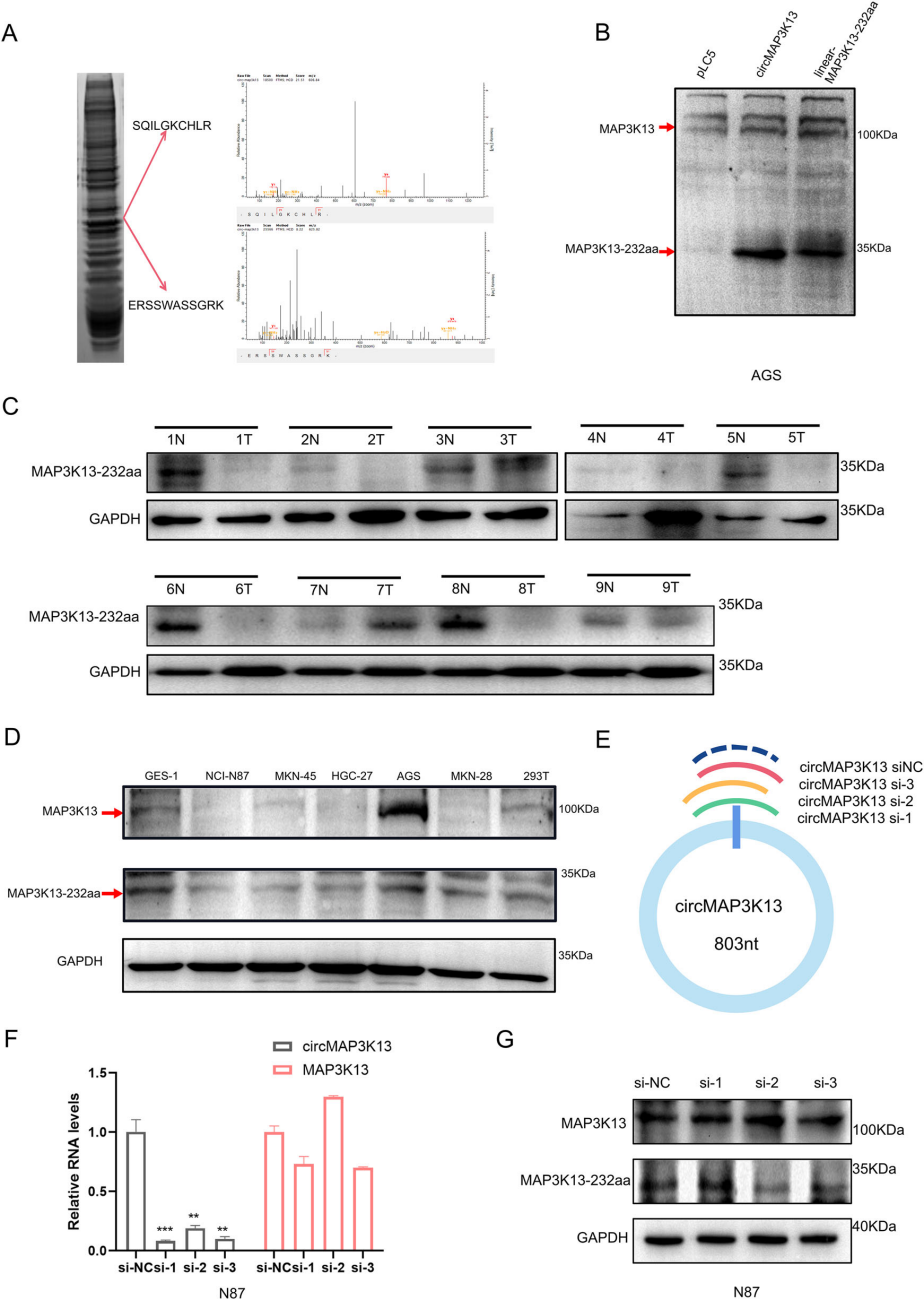
CircMAP3K13 encodes a novel protein, MAP3K13-232aa. **A** Mass spectrometry (IP-MS) following immunoprecipitation was conducted to identify the unique peptide of MAP3K13-232aa. **B** A commercial MAP3K13 antibody for MAP3K13 was used to detect the overexpressed MAP3K13-232aa and endogenous MAP3K13-232aa by immunoblotting. **C** MAP3K13-232aa expression level was analyzed in nine pairs of GC and paired adjacent normal tissues by western blot. N normal tissue, T tumor tissue. **D** Immunoblot analysis of MAP3K13-232aa across various cell lines, including GC cell lines (NCI-N87, MKN-45, HGC-27, AGS, MKN-28), a normal gastric epithelial cell line (GES-1), and 293 T cells. GAPDH served as the loading control. **E** Schematic of three siRNAs targeting circMAP3K13. **F** qPCR results of circMAP3K13 expression after transfection with si-1, si-2, and si-3. **G** Western blot analysis of MAP3K13-232aa protein as well as full-length MAP3K13 after circMAP3K13 siRNA transfection. GAPDH served as the loading control. Unless otherwise specified, statistical significance was assessed using a two-tailed Student’s t-test. *P < 0.05; **P < 0.01; ***P < 0.001; ****P < 0.0001.

To experimentally confirm the protein’s existence in cells, we transfected AGS cells with the circMAP3K13 plasmid and performed IP followed by mass spectrometry (IP-MS). A unique peptide specific to MAP3K13-232aa, and distinct from full-length MAP3K13, was identified in GC cells (Fig. 4A), validating the expression of this novel protein. The full-length MAP3K13 protein comprises 966 amino acids, and partially overlaps with MAP3K13-232aa between residues 233–426 (Supplementary Fig. 1D).

Using a commercial antibody that recognizes both full-length MAP3K13 and MAP3K13-232aa, we performed western blotting in AGS cells and detected both protein forms, confirming MAP3K13-232aa was endogenously expressed in GC cells (Fig. 4B). MAP3K13-232aa was further detected in GC and corresponding adjacent non-neoplastic tissues, being highly expressed in 88.9% (8/9) of adjacent normal tissues but relatively reduced in GC tissues, consistent with the qPCR findings (Figs. 4C, 2E). Western blot analysis also showed lower levels of MAP3K13-232aa in GC cell lines compared to the normal gastric epithelial cell line GES-1 (Fig. 4D).

To functionally validate the relationship between circMAP3K13 gene expression and MAP3K13-232aa, we designed three siRNAs targeting circMAP3K13 (Fig. 4E). RT-PCR and western blot analysis demonstrated that these siRNAs specifically reduced circMAP3K13 expression without affecting MAP3K13 mRNA levels (Fig. 4F, G, Supplementary Fig. 2A). These results confirmed that circMAP3K13 encodes the novel protein MAP3K13-232aa, and that siRNA-mediated knockdown of circMAP3K13 effectively reduces expression of the novel protein MAP3K13-232aa.

### MAP3K13-232aa inhibits the proliferation, migration, and invasion of GC cells

**Fig. 5:**
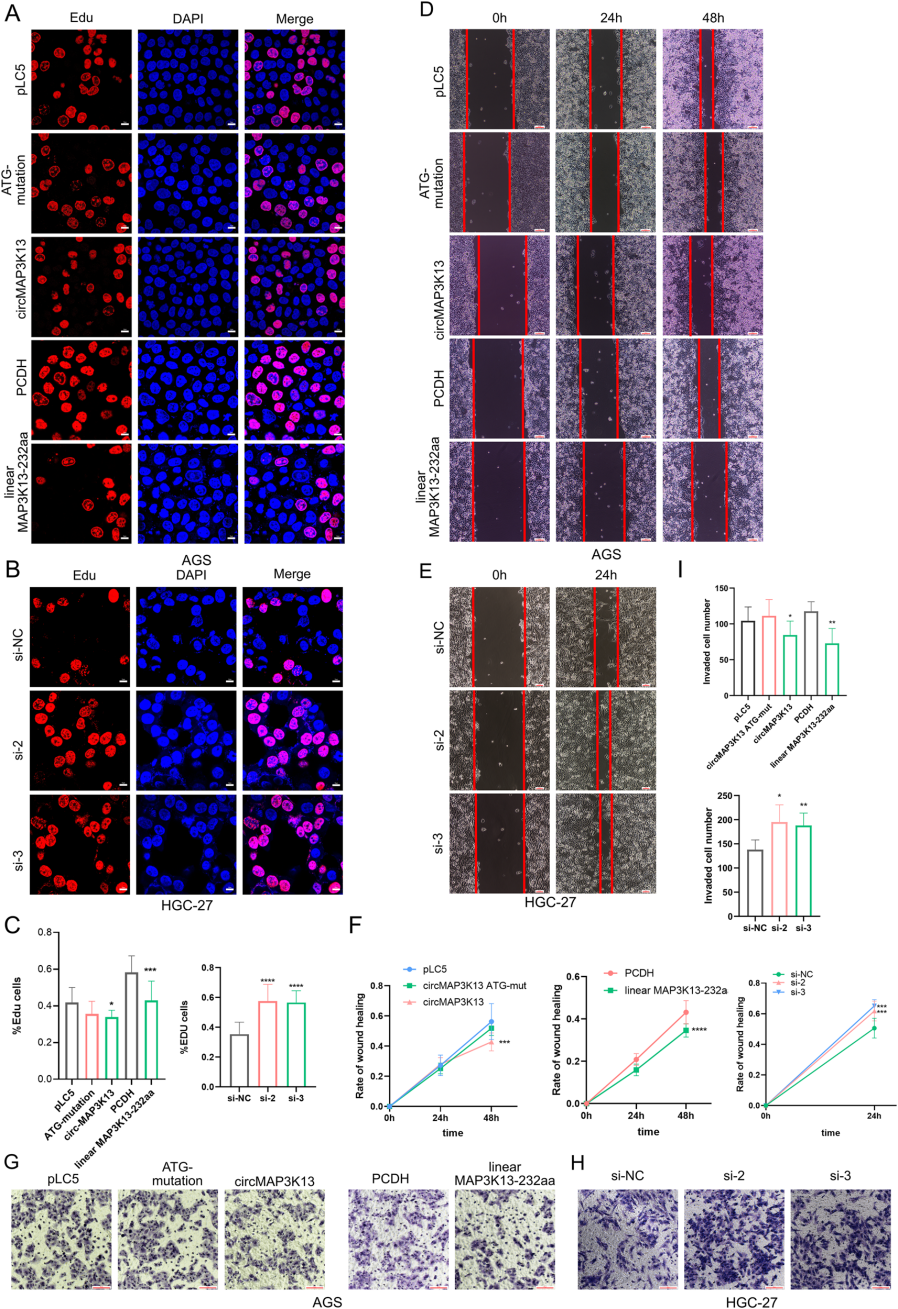
circMAP3K13 suppresses the proliferation and migration of GC cells. **A** Cell proliferation was determined by using EdU staining (red) after overexpression of MAP3K13-232aa in AGS cells and **B** after silencing circMAP3K13 with siRNAs transfection in HGC-27 cells. The nuclei are counterstained with DAPI (blue). Scale bar = 10 μm. **C** Quantification of EdU-positive cells from (**A**, **B**). **D** Wound healing assay was performed after circMAP3K13 and MAP3K13-232aa overexpression. Scale bar = 100 μm. **E** Wound healing assay was performed after knockdown of circMAP3K13 in HGC-27 cells. Scale bar = 200 μm. **F** Quantification of wound healing assays is shown. **G** Transwell assay was conducted after overexpressing MAP3K13-232aa in AGS cells. Scale bar = 100 μm. **H** Transwell assay was conducted after transfection of circMAP3K13-targeting siRNAs in HGC-27 cells. Scale bar = 100 μm. **I** Quantification of Transwell invasion assay results is shown. Unless otherwise specified, statistical significance was assessed using a two-tailed Student’s t-test. *P < 0.05; **P < 0.01; ***P < 0.001; ****P < 0.0001.

To investigate the biological function of circMAP3K13, AGS cells were transfected with five plasmids: a circMAP3K13 overexpression vector, an ATG-mutant circMAP3K13 construct, a linear MAP3K13-232aa expression vector, and two empty vector controls. Both circMAP3K13 and linear MAP3K13-232aa significantly suppressed AGS cell proliferation, as shown by decreased EdU incorporation and reduced colony formation rates (Fig. 5A, C, Supplementary Fig. 2B). By contrast, the ATG-mutant circMAP3K13 had no inhibitory effect, indicating that its tumor-suppressive activity is dependent on protein translation.

To further confirm the role of circMAP3K13, we transfected circMAP3K13-targeting siRNAs into HGC-27 cells. Knockdown of circMAP3K13 promoted GC cell proliferation (Fig. 5B, C), supporting its inhibitory role. Similarly, AGS cells overexpressing MAP3K13-232aa exhibited slower wound closure, suggesting reduced migratory capacity. Conversely, HGC-27 cells transfected with siRNAs (no. 2 and 3) showed significantly increased migration compared to cells transfected with siNC (Fig. 5D–F).

In transwell invasion assays, both AGS and HGC-27 cells overexpressing MAP3K13-232aa demonstrated decreased invasive ability compared to controls (Fig. 5G–I). Together, these results suggest that MAP3K13-232aa has a tumor-suppressive role in GC, inhibiting cell proliferation, migration, and invasion.

### MAP3K13-232aa directly binds the IKKα kinase domain and increases its kinase activity

**Fig. 6:**
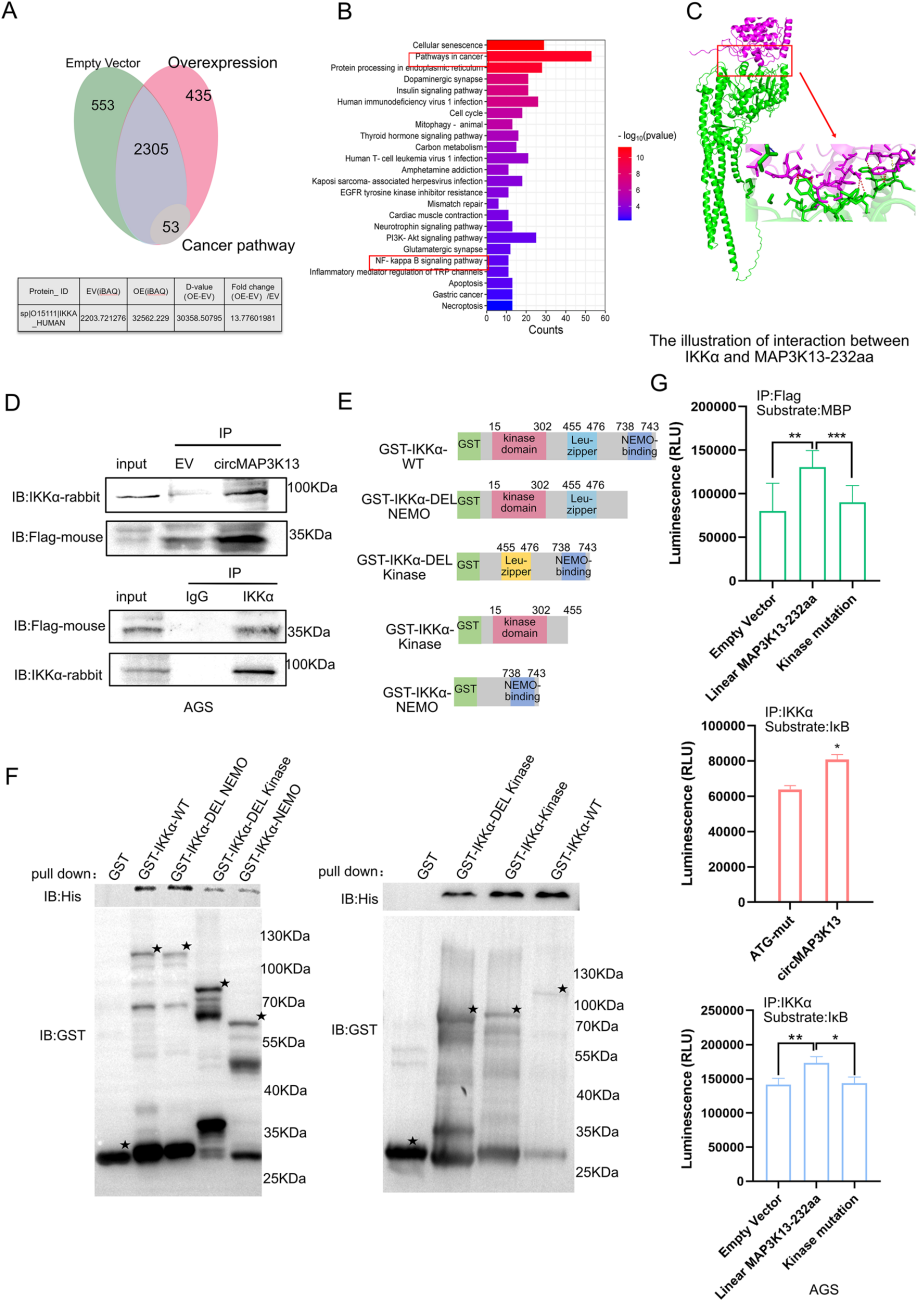
MAP3K13-232 directly binds to the kinase domain of IKKα. **A** Proteins interacting with MAP3K13-232aa were identified by immunoprecipitation followed by mass spectrometry (IP-MS). A total of 2305 proteins were pulled down by anti-flag antibody from lysates of cells transfected with circMAP3K13 or empty vector using an anti-FLAG antibody. Of these, 435 proteins were unique to the circMAP3K13 group. Differentially abundant proteins (fold change >2) were subjected to KEGG pathway enrichment analysis. **B** Fifty-three proteins with a fold change >13, including the IKKα, with fold changes >13 were enriched in cancer-related pathways; several enriched pathways identified in the enrichment analysis are shown. **C** The interaction between MAP3K13-232aa and IKKα was modeled using Alphafold3. IKKα is shown in green, and the purple structure is MAP3K13-232aa. Chemical interactions were visualized using PyMOL. Yellow dotted lines represent hydrogen bonds, red dotted lines indicate hydrophobic interactions, and blue dotted lines denote salt bridges. **D** Co-immunoprecipitation experiments were performed to detect the interaction between MAP3K13-232aa and IKKα. **E** Schematic of the five GST-tagged IKKα recombinant protein constructs used in the GST pull-down assay: GST-WT (full-length recombinant GST-IKKα); GST-IKKα-ΔNEMO (lacking the NEMO-binding region); GST-IKKα-ΔKinase (kinase domain of GST-IKKα was deleted); GST- IKKα-Kinase (only the kinase domain); GST-IKKα-NEMO (only included the NEMO-binding region of GST-IKKα and lacked the kinase domain, lacking the kinase and leucine zipper regions). **F** Five “GST-tagged constructs and a His-tagged MAP3K13-232aa construct were expressed in E. coli BL21(DE3). Purified proteins were incubated to form immunocomplexes, followed by western blotting using anti-GST and anti-His antibodies. **G** AGS cells were transfected with ATG-mutation, circMAP3K13, wild-type circMAP3K13, empty vector (PCDH), linear MAP3K13-232aa, or kinase-dead MAP3K13-232aa. Whole-cell lysates were collected and subjected to immunoprecipitation for kinases enrichment. In vitro kinase assay was performed. Upper panel: IP FLAG; middle and lower panels: IP IKKα. Substrates used were IκB for IKKα and MBP for MAP3K13-232aa. Unless otherwise specified, statistical analysis was performed using a two-tailed Student’s t-test. *P < 0.05; **P < 0.01; ***P < 0.001.

To investigate how MAP3K13-232aa regulates GC progression, we first performed IP-MS to identify its interacting proteins. AGS cells were transfected with MAP3K13-232aa–FLAG or an empty vector, and FLAG-tagged proteins were pulled down using an anti-FLAG antibody. Differential protein expression analysis of the IP-MS results revealed 53 candidate interactors involved in the cancer-related pathways, including IKKα (Fig. 6A). IKKα phosphorylates IκB to activate the NF-κB signaling pathway [14]. Consequently, pathway enrichment analysis of the IP-MS data also indicated significant enrichment of NF-κB signaling pathway components (Fig. 6B), suggesting that MAP3K13-232aa may exert its effects through this pathway.

To further explore this interaction, we used AlphaFold3 to model the 3D binding structure of the MAP3K13-232aa–IKKα complex. The model predicted a direct interaction between MAP3K13-232aa and the α-helix region of IKKα (Fig. 6C). To identify interacting residues at the protein-protein interface, we analyzed the interaction using PyMOL, focusing on residues within a 6 Å distance. Hydrogen bonds (≤4 Å), hydrophobic interactions (≤4 Å), and salt bridges (≤5 Å) were visualized. Notable interactions included VAL124 (MAP3K13-232aa) with PHE181 (IKKα), MET79 with GLN226, ASP125 with THR182, and ILE130 with LEU186. The interaction between MAP3K13-232aa and IKKα was confirmed by co-immunoprecipitation (Co-IP), in which anti-FLAG and anti-IKKα antibodies successfully co-precipitated both proteins (Fig. 6D).

To determine whether MAP3K13-232aa binds directly to IKKα and to map the binding domain, GST pull-down assays were performed. IKKα contains three main functional domains: the protein kinase domain, the leucine zipper region, and the NEMO-binding region. We therefore generated five GST-tagged constructs: GST–wild-type IKKα (full length); GST–ΔNEMO (deletion of the NEMO-binding region); GST–Δkinase (deletion of the kinase domain); GST–kinase (encoding the functional kinase domain of IKKα); and GST NEMO (which only included the NEMO-binding region) (Fig. 6E). The sequence of MAP3K13-232aa was cloned into a His-tag vector to express the MAP3K13-232aa-His recombinant protein. Pull-down assays showed that MAP3K13-232aa directly interacted with IKKα, specifically at the kinase domain (Fig. 6F, Supplementary Fig. 2C).

Because MAP3K13-232aa originates from the kinase domain of the full-length MAP3K13 protein (Supplementary Fig. 1E), we hypothesized that it may possess intrinsic kinase activity and modulate IKKα activity via a direct interaction. To test this hypothesis, we generated a kinase-defective mutant of MAP3K13-232aa by substituting the conserved aspartic acid residue (D) with alanine (A) (GAT → GCT), a critical change known to disrupt enzymatic activity [28]. Using Myelin Basic Protein as a substrate, we found that wild-type MAP3K13-232aa exhibited kinase activity, whereas the D-to-A mutant did not (Fig. 6G), supporting our hypothesis. Moreover, overexpression of circMAP3K13 or MAP3K13-232aa markedly increased the IKKα kinase activity (Fig. 6G) and the phosphorylation of IKKα/β (Supplementary Fig. 2D). By contrast, the kinase-dead MAP3K13-232aa mutant failed to enhance IKKα kinase activity (Fig. 6G middle and lower) and IKKα/β phosphorylation (Supplementary Fig. 2D), suggesting that MAP3K13-232aa possibly harbors functional kinase activity and regulates activity of IKKα/β by directly binding to its kinase domain.

### MAP3K13-232aa activates the NF-κB signaling pathway

To test whether MAP3K13-232aa regulates the NF-κB signaling pathway by enhancing IKKα kinase activity, we overexpressed circMAP3K13 and MAP3K13-232aa in AGS cells and silenced circMAP3K13 in NCI-N87 cells. Immunoblotting was then performed to assess NF-κB pathway activation. Transfected cells were treated with 20 ng/mL TNF-α for 5 min to activate NF-κB pathway before sample collection.

**Fig. 7:**
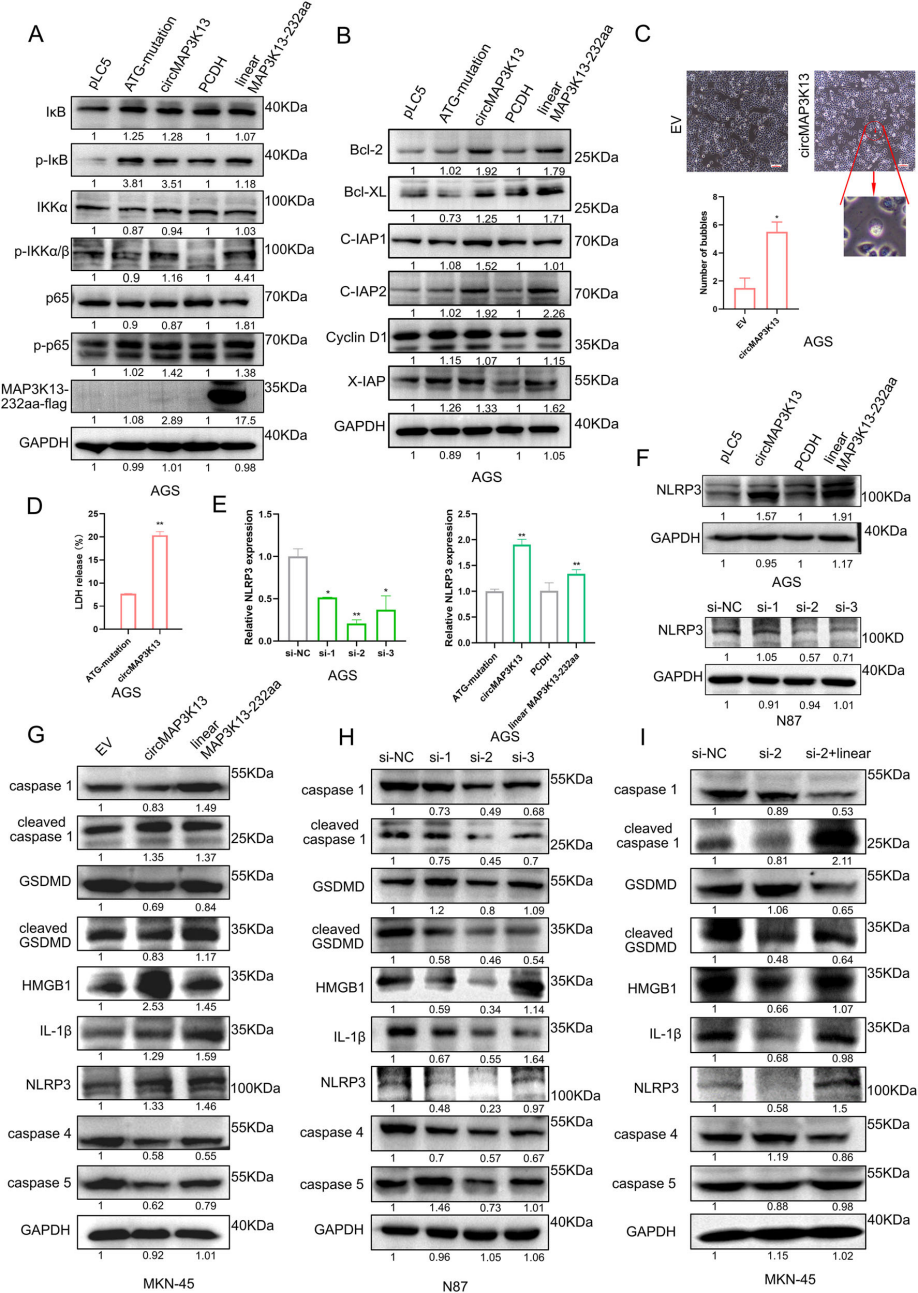
MAP3K13-232aa enhances cisplatin-induced pyroptosis through activating the NF-κB signaling pathway. **A** Western blot analysis of AGS cells transfected with circMAP3K13 overexpression plasmids. Cells were treated with 20 ng/mL TNFα for 5 min prior to protein collection. NF-κB signaling pathway-related proteins were detected by western blot. **B** Detection of NF-κB downstream target proteins in AGS cells transfected with circMAP3K13 overexpression plasmids. **C** AGS cells transfected with either an empty vector or circMAP3K13 overexpression plasmid circMAP3K13 groups were treated with LPS (2 μg/mL) for 4 h and nigericin (20 μM) for 1 h. Red arrows indicate protrusive vesicles characteristic of pyroptosis. **D** LDH levels in the culture supernatant were measured in AGS cells under the same treatment conditions to evaluate the extent of pyroptosis. **E** NLRP3 mRNA expression was assessed in AGS cells after induction of pyroptosis. **F** Western blot analysis was used to detect NLRP3 protein expression following pyroptosis induction after circMAP3K13 overexpression and knockdown. Western blot analysis of pyroptosis-related proteins in MKN-45 (**G**) or N87 (**H**) cells, which were treated with cisplatin (1 μg/mL) for 48 h. **I** Rescue assay showing the effect of MAP3K13-232aa overexpression following circMAP3K13 knockdown on pyroptosis-associated protein expression. Unless otherwise specified, statistical analysis was performed using a two-tailed Student’s t-test. *P < 0.05; **P < 0.01; ***P < 0.001; ****P < 0.0001.

In AGS cells overexpressing MAP3K13-232aa, IκB phosphorylation was elevated and p65 was activated (Fig. 7A). By contrast, knockdown of circMAP3K13 in NCI-N87 cells reduced the phosphorylation of both IκB and p65 (Supplementary Fig. 2E). Interestingly, the ATG-mutant circMAP3K13 also led to increased phosphorylation of IκB and p65, but not IKKα, suggesting that circMAP3K13 may activate NF-κB signaling partly through mechanisms independent of MAP3K13-232aa. The increase in phosphorylated (p)-IKKα further supports that MAP3K13-232aa directly promotes IKKα kinase activity and thereby stimulates NF-κB signaling.

Next, we evaluated the expression of downstream target genes of the NF-κB signaling pathway, including *Bcl-XL*, *Bcl-2*, *XIAP*, and *c-IAP1*. Immunoblot analysis showed that overexpression of circMAP3K13 and MAP3K13-232aa upregulated these targets, whereas circMAP3K13 siRNA-mediated knockdown reduced their expression (Fig. 7B, Supplementary Fig. 2F). The ATG-mutant also increased target gene expression, although to a lesser extent than MAP3K13-232aa, further supporting that circMAP3K13 can activate NF-κB signaling partially through mechanisms independent of its protein product. Together, these findings indicate that MAP3K13-232aa enhances NF-κB signaling pathway activity by promoting IKKα kinase activity.

### MAP3K13-232aa enhances chemotherapy drug-induced pyroptosis

Pyroptosis is a form of regulated cell death that can inhibit tumor cell proliferation, migration, and invasion, but may also help to create a tumor-promoting microenvironment. The specific role of pyroptosis depends on factors such as the tumor cell type, the tumor microenvironment, and the nature of the pyroptotic stimuli [29]. Studies have shown that the NF-κB signaling pathway facilitates pyroptosis by modulating the transcription of NLRP3 [16]. To explore whether circMAP3K13 induces pyroptosis, we treated AGS cells with LPS and nigericin and transfected them with either circMAP3K13 or an empty vector. Compared to controls, AGS cells overexpressing circMAP3K13 exhibited more pronounced plasma membrane blebbing, a hallmark of pyroptosis (Fig. 7C). Additionally, these cells released higher levels of LDH, further indicating increased pyroptotic activity (Fig. 7D).

Given that the NF-κB signaling pathway may be activated by MAP3K13-232aa, we hypothesized that this protein promotes pyroptosis by upregulating NF-κB downstream target NLRP3. To test this, MAP3K13-232aa was overexpressed in AGS cells treated with LPS (4 h) and nigericin (1 h). Overexpression of circMAP3K13 or MAP3K13-232aa led to increased NLRP3 mRNA and protein levels, whereas circMAP3K13 knockdown resulted in reduced NLRP3 expression. (Fig. 7E, F), supporting the role of MAP3K13-232aa in positively regulating NLRP3.

We then assessed expression of pyroptosis-associated proteins in AGS and NCI-N87 cells. Upon treatment with LPS and nigericin, MAP3K13-232aa overexpression increased levels of cleaved caspase**-**1, cleaved GSDMD, and IL-1β, indicating enhanced pyroptosis in AGS cells (Supplementary Fig. 2G). CircMAP3K13 knockdown resulted in decreased levels of pyroptosis associated proteins, suggesting the stimulative role of circMAP3K13 in regulating pyroptosis (Supplementary Fig. 2H).

Chemo-resistance is a common issue in cancer treatment [30], with patients exhibiting drug resistance often facing poor clinical outcomes. Pyroptosis has been observed in response to chemotherapeutic agents such as paclitaxel, cisplatin, etoposide, and doxorubicin, primarily through cleavage of GSDMD and GSDME by executioner caspase-3 [31, 32]. We thus hypothesized that MAP3K13-232aa enhances chemotherapy-induced pyroptosis. To test this hypothesis, we analyzed pyroptosis markers in MKN-45 and NCI-N87 cells treated with cisplatin (1 μg/mL). In MKN-45 cells, overexpression of circMAP3K13 or MAP3K13-232aa resulted in upregulation of NLRP3, cleaved caspase-1, and cleaved GSDMD (Fig. 7G). By contrast, circMAP3K13 knockdown in NCI-N87 cells led to reduced expression of these proteins (Fig. 7H). A rescue experiment further demonstrated that MAP3K13-232aa overexpression restored pyroptosis following circMAP3K13 knockdown (Fig. 7I).

Collectively, these findings demonstrate that MAP3K13-232aa promotes pyroptosis in GC cells by activating the NF-κB signaling pathway and enhances chemotherapy-induced pyroptosis.

### MAP3K13-232aa reduces GC tumorigenicity in vivo

**Fig. 8:**
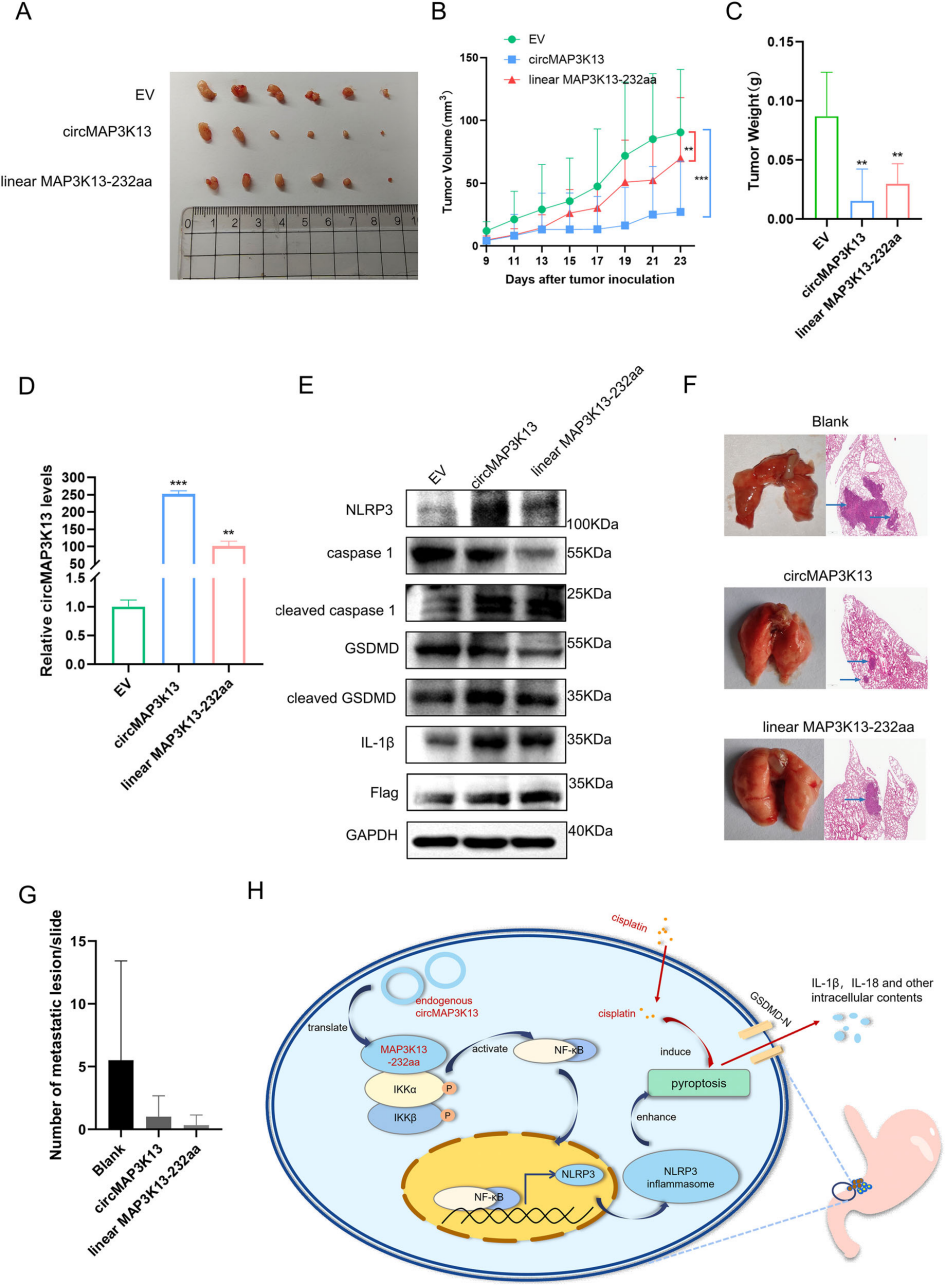
Overexpression of circMAP3K13 overexpression curbs tumor growth in vivo*.* **A** Tumorigenic models were established by subcutaneous injection of nude mice with 1 × 10⁶ MKN-45 cells which were stably transfected with circMAP3K13, MAP3K13-232aa, or empty vector. **B**, **C** Tumor volume was measured at regular intervals in each group. **C** Tumor weight was assessed at the endpoint. **D** Quantitative PCR and western blot analyses were performed on tumor tissues to determine the expression levels of circMAP3K13 and MAP3K13-232aa. **E** Pyroptosis-related proteins were detected in tumor lysates to evaluate pyroptotic activity. **F** Hematoxylin and eosin staining to identify metastases in the lungs of nude mice. **G** Quantification of metastatic lung nodules in each group. **H** Schematic model of the proposed mechanism: circMAP3K13 encodes MAP3K13-232aa, which binds to the kinase domain of IKKα to activate the NF-κB signaling and promote cisplatin-induced pyroptosis. Unless otherwise specified, statistical analysis was performed using a two-tailed Student’s t-test. **P < 0.01; ***P < 0.001.

To validate the tumor-suppressive role of MAP3K13-232aa in vivo, we established stably transfected MKN-45 cell lines overexpressing circMAP3K13, linear MAP3K13-232aa, or an empty vector. These cells were subcutaneously injected into nude mice to generate xenograft tumors. Mice injected with MAP3K13-232aa–overexpressing cells developed tumors but with markedly reduced tumor volumes and weights compared to controls (Fig. 8A–C). Quantitative PCR and western blotting confirmed successful overexpression of MAP3K13-232aa in the xenograft tissues (Fig. 8D, E).

We then analyzed protein extracts from the tumors for pyroptosis markers. Expression of cleaved GSDMD, cleaved caspase-1, and IL-1β was elevated in tumors overexpressing MAP3K13-232aa, indicating enhanced pyroptosis in vivo (Fig. 8E). To assess the impact on metastasis, lung metastasis models in nude mice were established by tail vein injection of the same stably transfected cell lines. H&E staining revealed that mice injected with MAP3K13-232aa–overexpressing cells had fewer metastatic tumor nodules in the lungs compared to the control group (Fig. 8F, G). These results demonstrate that MAP3K13-232aa acts as a tumor suppressor in vivo, consistent with our in vitro findings.

In summary, we found that circMAP3K13, which is downregulated in GC tissues, encodes MAP3K13-232aa, which activates the NF-κB signaling pathway by directly binding to the kinase domain of IKKα. MAP3K13-232aa promotes pyroptosis in GC cells by upregulating the expression of NLRP3, and enhances cisplatin-induced pyroptosis. Pyroptosis, in turn, triggers membrane rupture and the release of intracellular contents, ultimately leading to cell death and suppression of GC cell proliferation, metastasis, and invasion, and metastasis (Fig. 8H).

## Discussion

Over the past several decades, substantial efforts have been dedicated to understanding the pathogenesis of GC to reduce its high mortality rate. Recently, abnormally expressed circRNAs have been implicated in tumorigenesis and cancer metastasis across multiple cancer types [33, 34], including GC [35]. In most GC studies to date, circRNAs have been shown to regulate cancer development by interacting with proteins or microRNAs. For example, circ_CEA is highly expressed in GC tissues and promotes tumor progression by forming a scaffold with p53 and CDK1 to suppress apoptosis [36]. In addition, some circRNAs regulate GC cell proliferation and cell cycle progression via their encoded novel proteins. GSPT1-238aa, encoded by circGSPT1, is expressed at low levels in GC tissues and suppresses carcinogenesis by inhibiting autophagy [37], while circAXIN1, which is highly expressed circRNA in GC cells, encodes the oncogenic protein AXIN1-295aa, which competitively binds to APC to enhance Wnt signaling activity [25]. Identifying and characterizing differentially expressed circRNAs in GC, therefore, holds great potential for identifying and developing new diagnostic and therapeutic approaches.

In this study, we used RNA-seq and Ribo-seq to screen for differentially expressed genes in GC and identified circMAP3K13, derived from a gene known to contribute to GC progression [26]. CircMAP3K13 was downregulated in GC tissues compared with normal tissues, and its expression was negatively correlated with tumor stage, infiltration depth, and lymph node metastasis. These findings suggest a tumor-suppressive role for circMAP3K13 and its potential as a therapeutic target in GC tumors. We further showed that circMAP3K13 encodes a novel protein, MAP3K13-232aa, which inhibits GC development. Gain- and loss-of-function experiments revealed that MAP3K13-232aa suppressed the proliferation and migration of AGS and NCI-N87 cells in vitro. Consistent with these findings, in vivo experiments confirmed that MAP3K13-232aa significantly inhibited tumor growth and lung metastasis in mouse models.

The NF-κB pathway regulates inflammation, tumorigenesis, and immune responses in eukaryotic cells [38]. Recent studies in rat models and cultured cells have reported that circRNAs can modulate NF-κB signaling to mediate disease processes. For instance, Circ-Sirt limits nuclear translocation of NF-κB p65 to prevent vascular inflammation [39], while circPPM1F promotes β-cell apoptosis in a mouse model of type 1 diabetes by inducing the NF-κB pathway [40]. In our study, co-IP and GST pull-down assays demonstrated that MAP3K13-232aa directly binds to the kinase domain of IKKα. We also confirmed that MAP3K13-232aa promotes IKKα phosphorylation and increases the expression of downstream NF-κB target genes. Functionally, this activation suppressed GC cell proliferation, migration, and invasion, suggesting that MAP3K13-232aa inhibits GC progression through activation of the NF-κB pathway.

To further investigate this mechanism, we focused on the relationship between NF-κB signaling and pyroptosis—a form of inflammatory programmed cell death. Prior research has shown that NF-κB plays a key role in initiating pyroptosis by promoting the expression of genes such as *NLRP3, caspase-1, caspase-11, TLR4, IRF7*, and *GSDMD* [41]. These findings prompted us to examine whether circMAP3K13 and MAP3K13-232aa influence pyroptosis in GC. Emerging evidence has revealed that pyroptosis is modulated by non-coding RNAs and plays a role in the pathogenesis of diseases such as atherosclerosis [42], skeletal muscle ischemic injury [43], alcoholic liver disease [44], and GC [45]. For instance, circHIPK3 inhibits pyroptosis and protects skeletal muscle cells from ischemic damage by promoting FOXO3a expression [43]. circRNAs and their encoded proteins are thus increasingly recognized [46], but their role in tumor cell pyroptosis remains poorly understood.

We found that AGS cells overexpressing circMAP3K13 displayed increased membrane blebbing following LPS and nigericin treatment—consistent with pyroptotic morphology [47]. Western blotting confirmed that MAP3K13-232aa enhanced the expression of pyroptosis-related proteins, including cleaved caspase-1, GSDMD, and IL-1β, indicating that it may suppress GC progression through pyroptosis. In vitro and in vivo assays further demonstrated that MAP3K13-232aa inhibited GC cell proliferation and migration, reinforcing the tumor-suppressive function of pyroptosis mediated by circMAP3K13.

Cisplatin remains the primary chemotherapeutic drug for advanced GC [48], but resistance to cisplatin is common and often leads to poor clinical outcomes. Interestingly, recent studies have shown that cisplatin can induce pyroptosis by triggering caspase-3–mediated cleavage of GSDME in cancer cells [31]. In our experiments, MAP3K13-232aa enhanced GSDMD-mediated pyroptosis both in vitro and in vivo and sensitized GC cells to cisplatin. Thus, combined with cisplatin, circMAP3K13 might be useful for overcoming resistance and and improve therapeutic efficacy in advanced GC.

Although manipulating pyroptosis represents a promising anti-cancer strategy, translating this into clinical practice remains challenging. While the molecular mechanisms of cell death pathways such as apoptosis, necroptosis, and pyroptosis are well established [49], their physiological roles in modulating the tumor microenvironment remain poorly understood, and it can be difficult to correlate a particular type of cell death, such as pyroptosis, with a particular result. Tumor cells experience various, occasionally overlapping, types of cell death [50]. Suppressing one form may merely favor another. This complexity makes it difficult to predict outcomes and necessitates further research into agents that can modulate pyroptosis in combination with other therapies.

Although numerous studies have shown that circRNAs regulate tumor growth and progression, clinical translation remains difficult due to their low endogenous expression. Encouragingly, circRNAs can be synthesized in vitro by chemical and enzymatic strategies [51, 52]. For example, it has T4 RNA ligase 2, without a splint, enhances the circularization yield in vitro [53]. Such developments raise the possibility of generating artificial circMAP3K13 for therapeutic use in GC. Based on our data, we propose that when combined with conventional chemotherapy, such as cisplatin, circMAP3K13 or MAP3K13-232aa could improve treatment efficacy for drug-resistant patients with GC.

In summary, our study provides evidence that circMAP3K13 encodes a functional protein, MAP3K13-232aa, which activates NF-κB signaling, promotes pyroptosis, and suppresses tumor progression in GC. circMAP3K13 might be a potential therapeutic target that represses the proliferation and invasion of GC cells by promoting pyroptosis to potentially increase the survival rates of GC patients.

## Supplementary information

Supplementary Figure 1

Supplementary Figure 2

Supplementary figure legend

Table 1

Table 2

western blot original blot

## Data Availability

The data generated in this study are available within the article and its supplementary data files.
