# Supplementary material for: MAP3K13-232aa encoded by circMAP3K13 enhances cisplatin-induced pyroptosis by directly binding to IKKα in gastric adenocarcinoma
Source: Cell Death Dis. 2025 Sep 1;16(1):667. doi: 10.1038/s41419-025-07991-5 (PMC12402440; doi:10.1038/s41419-025-07991-5)
Supplement: Supplementary file 3 — Supplementary figure legend [file 41419_2025_7991_MOESM3_ESM.docx]

**Supplementary Fig. 1**

**A.** Differential expression patterns of MAP3K13 across various cancer types and matched normal tissues (including gastric tissue) were analyzed using the GEPIA2 database (http://gepia2.cancer-pku.cn/#general). **B.** Protein interaction networks associated with MAP3K13 were visualized using STRING software. **C.** The presence of circMAP3K13 in AGS and MKN-45 cell lines was verified by RT-PCR using divergent primers, with both cDNA and genomic DNA templates serving as PCR substrates. **D.** Subcellular localization of circMAP3K13 was determined by fluorescence in situ hybridization (FISH) using sequence-specific probes, following both circMAP3K13 overexpression and knockdown experimental conditions. **E.** MAP3K13-232aa contains a sequence overlap (amino acids 233-426) with full-length MAP3K13, while the yellow-highlighted region represents a unique peptide sequence. An anti-MAP3K13 antibody specifically recognizes the 1-194aa epitope within MAP3K13-232aa.

**Supplementary Fig. 2**

**A.** qPCR was performed to assess expression levels of circMAP3K13 and MAP3K13 in MKN-45 and HGC-27 cells following siRNA-mediated knockdown of circMAP3K13. **B.**Colony formation capacity of AGS cells was evaluated after transfection with circMAP3K13 overexpression vectors or MAP3K13-232aa expression constructs. **C.** Recombinant proteins were affinity-purified using HisSep™ Ni-NTA Agarose Resin and GSTSep™ Glutathione Agarose Resin. The left panel of the SDS-PAGE gel shows purified MAP3K13-232aa-His fusion proteins, while the right panel displays GST-tagged proteins: GST, GST-IKKα, GST-ΔNEMO, GST-ΔKinase, and GST-NEMO. Red asterisks (*) indicate positions of target proteins. **D.** Phosphorylation status of IKKα/β (p-IKKα/β) was analyzed by immunoblotting in AGS cells expressing wild-type MAP3K13-232aa or kinase-dead MAP3K13-232aa. **E.** Western blot analysis HGC-27 cells transfected with siRNAs targeting circMAP3K13. Cells were treated with 20 ng/mL TNFα for 5 minutes prior to protein collection. NF-κB signaling pathway-related proteins were detected by western blot. **F.** Detection of NF-κB downstream target proteins in HGC-27 cells transfected with circMAP3K13-targeting siRNAs. **G&H.**Pyroptosis was induced in AGS and N87 cells through sequential treatment with LPS (2 μg/mL, 4 h) and nigericin (20 μM, 1 h). Pyroptosis-related protein expression was assessed under: circMAP3K13/MAP3K13-232aa overexpression in AGS cells and circMAP3K13 knockdown in N87 cells.
