# Supplementary material for: MAP3K13-232aa encoded by circMAP3K13 enhances cisplatin-induced pyroptosis by directly binding to IKKα in gastric adenocarcinoma
Source: Cell Death Dis. 2025 Sep 1;16(1):667. doi: 10.1038/s41419-025-07991-5 (PMC12402440; doi:10.1038/s41419-025-07991-5)

Fig.3C

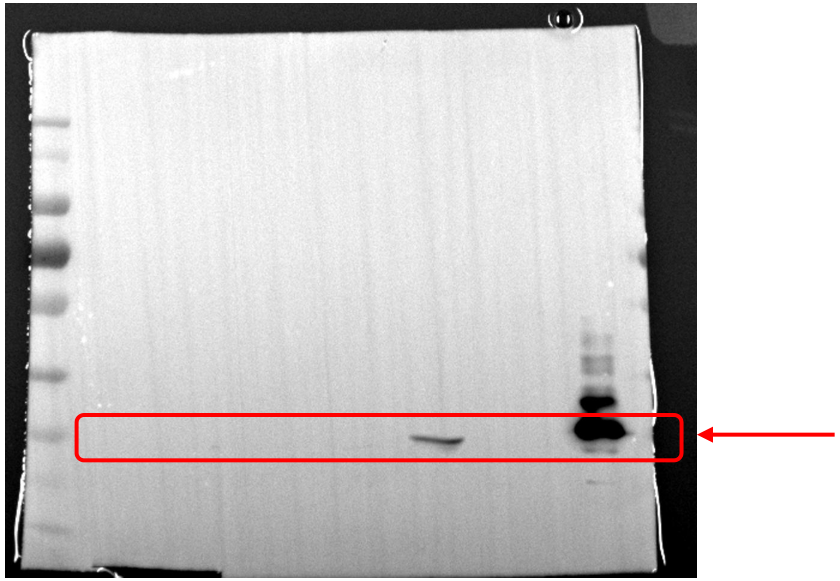

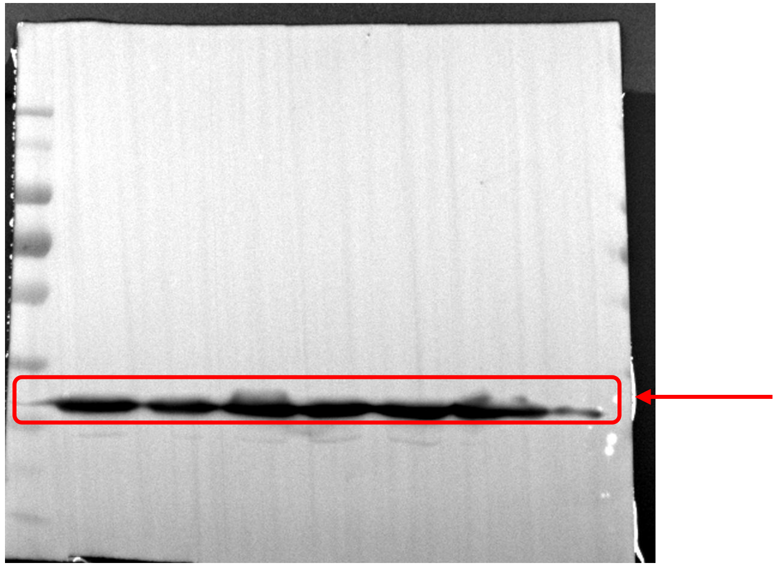

Fig.4B

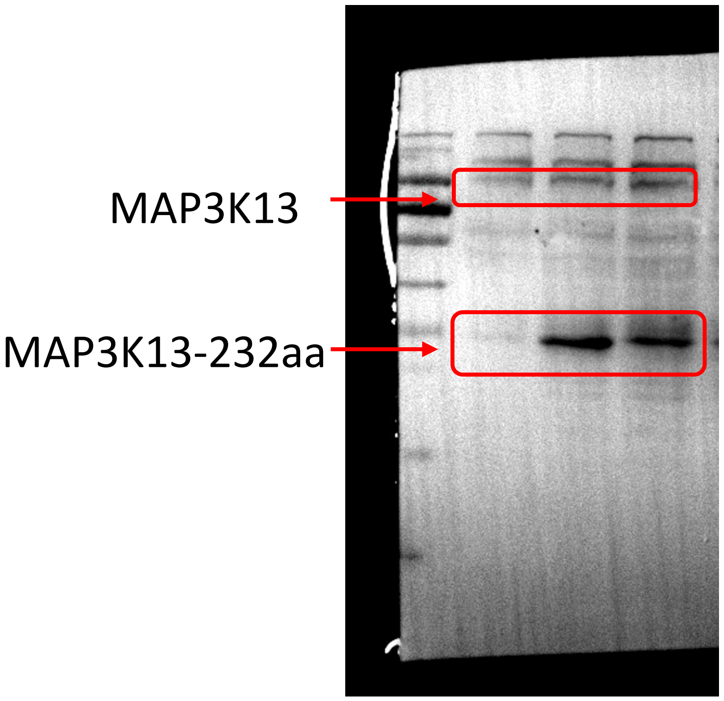
MAP3K13
MAP3K13-232aa

Fig.4C

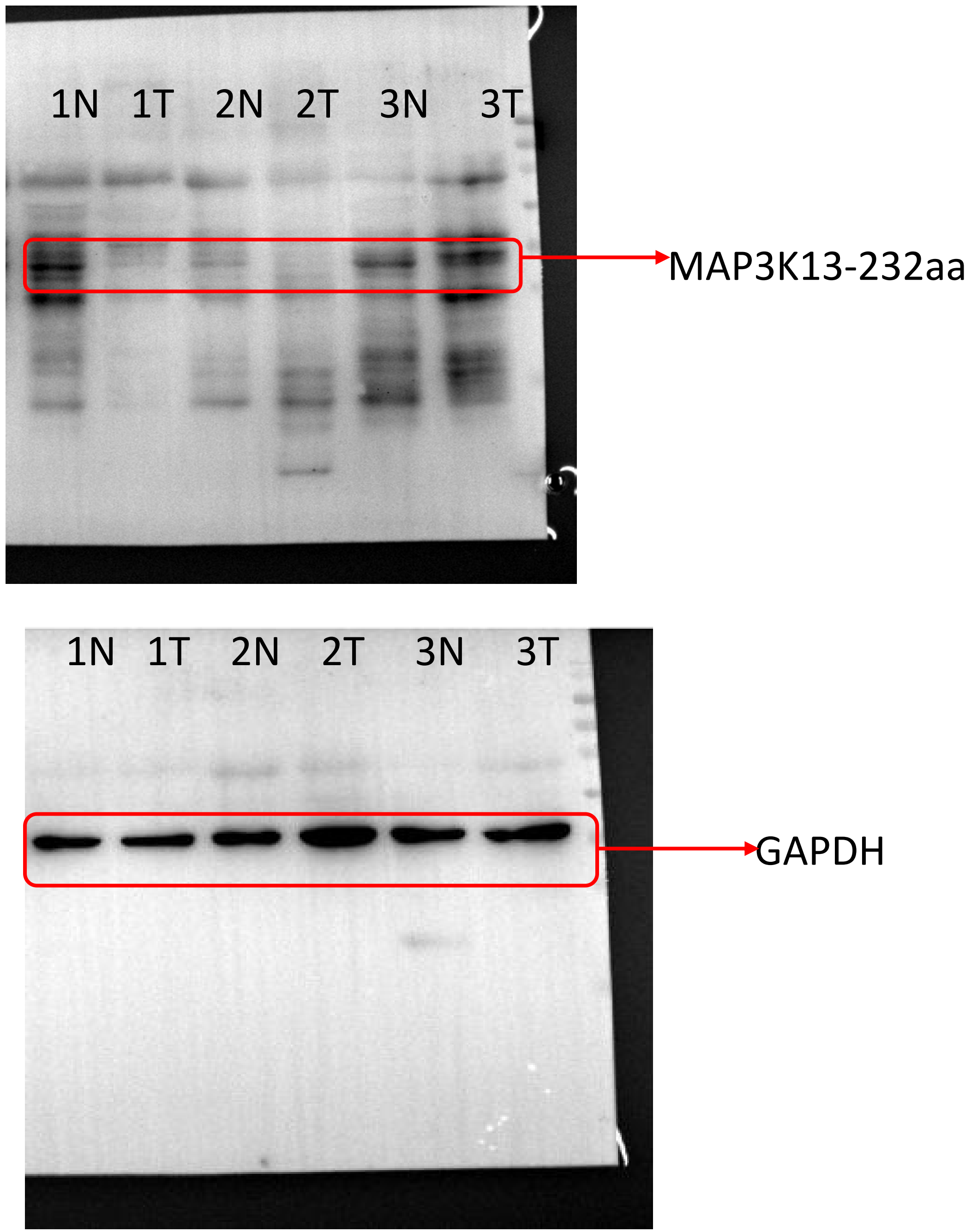
1N 1T 2N 2T 3N 3T
MAP3K13-232aa
1N 1T 2N 2T 3N 3T
GAPDH

Fig.4C

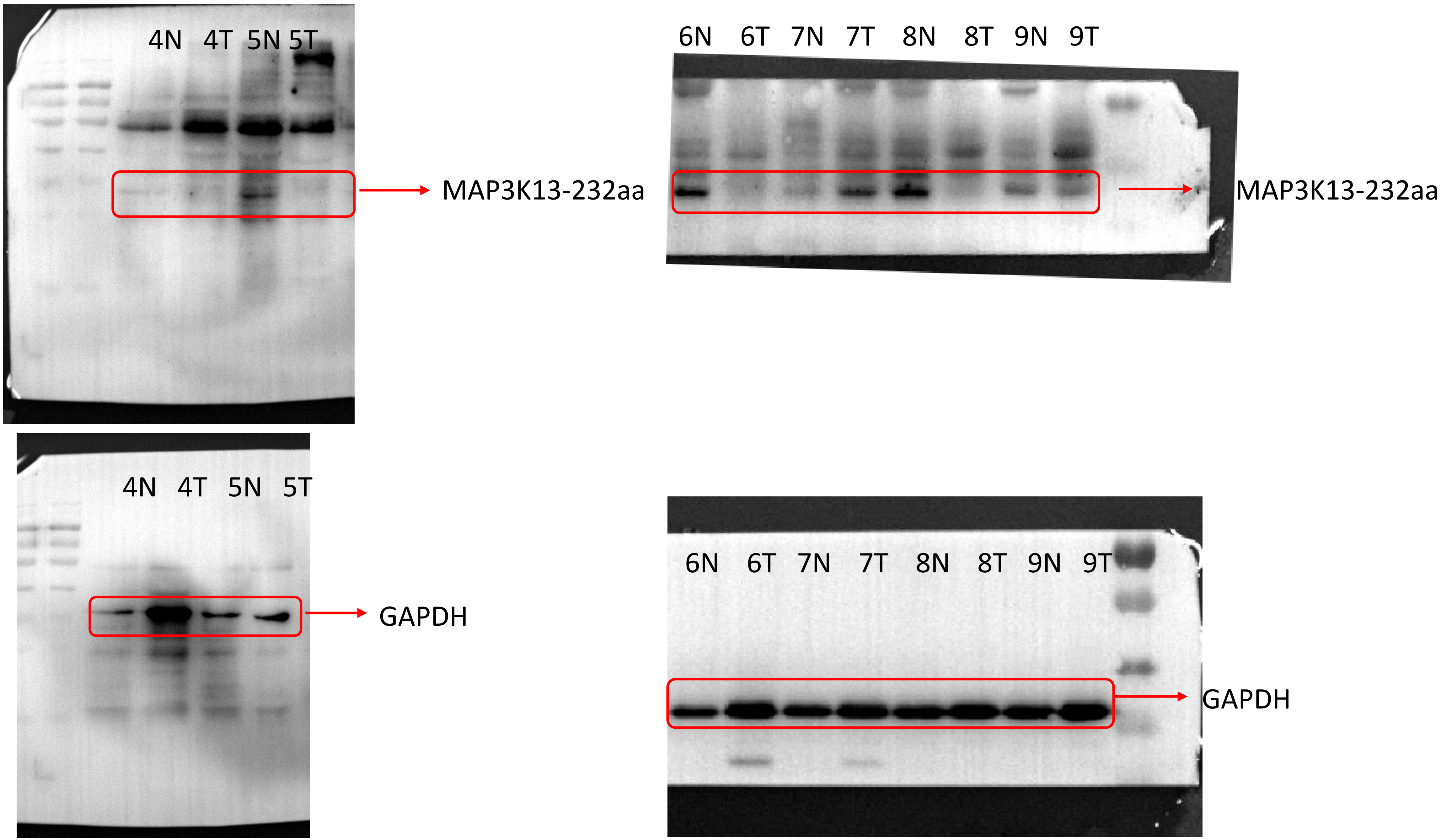

4N 4T 5N 5T
MAP3K13-232aa
6N 6T 7N 7T 8N 8T 9N 9T
MAP3K13-232aa
4N 4T 5N 5T
GAPDH
6N 6T 7N 7T 8N 8T 9N 9T
GAPDH

Fig.4D

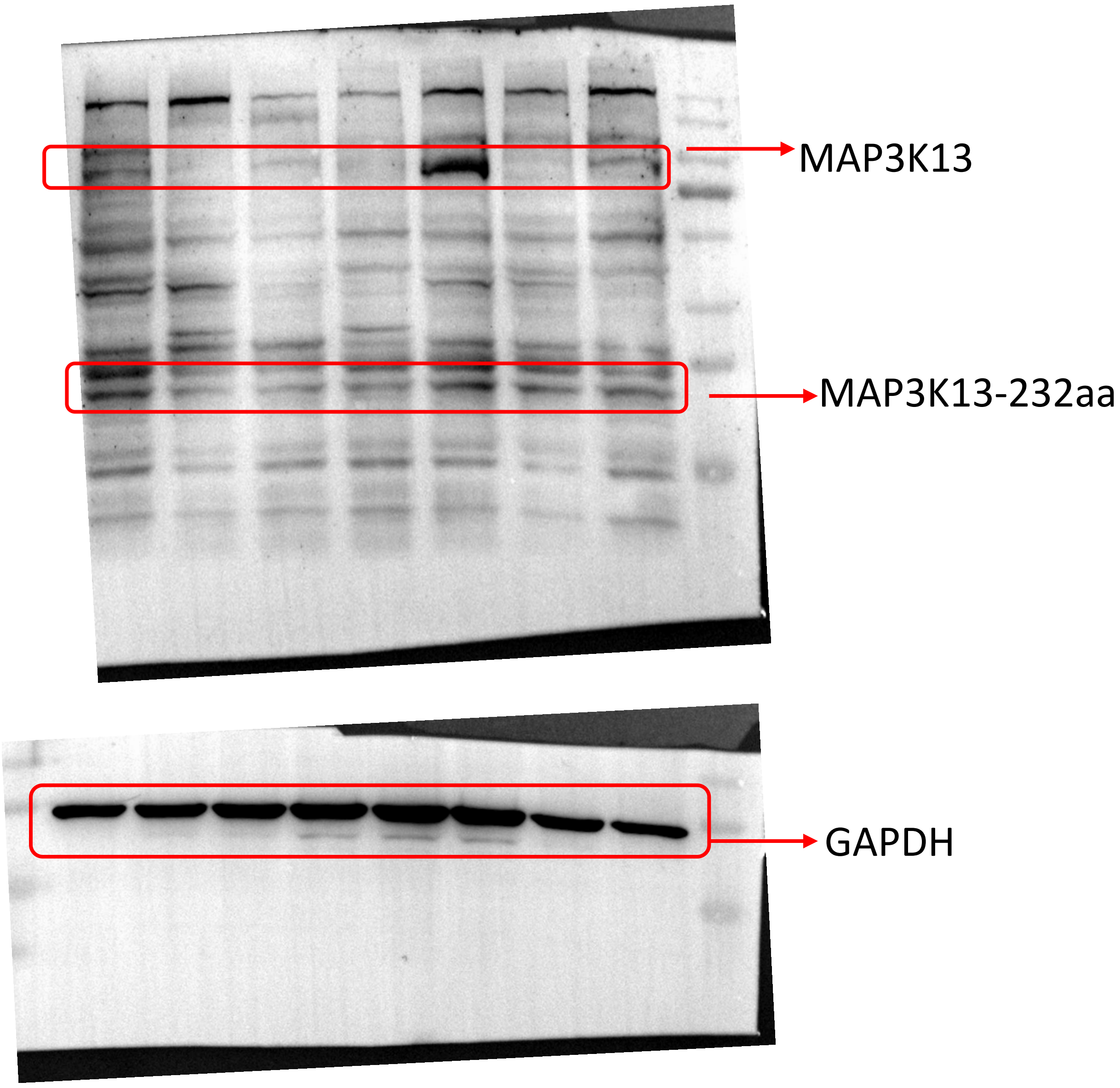

Fig.4G

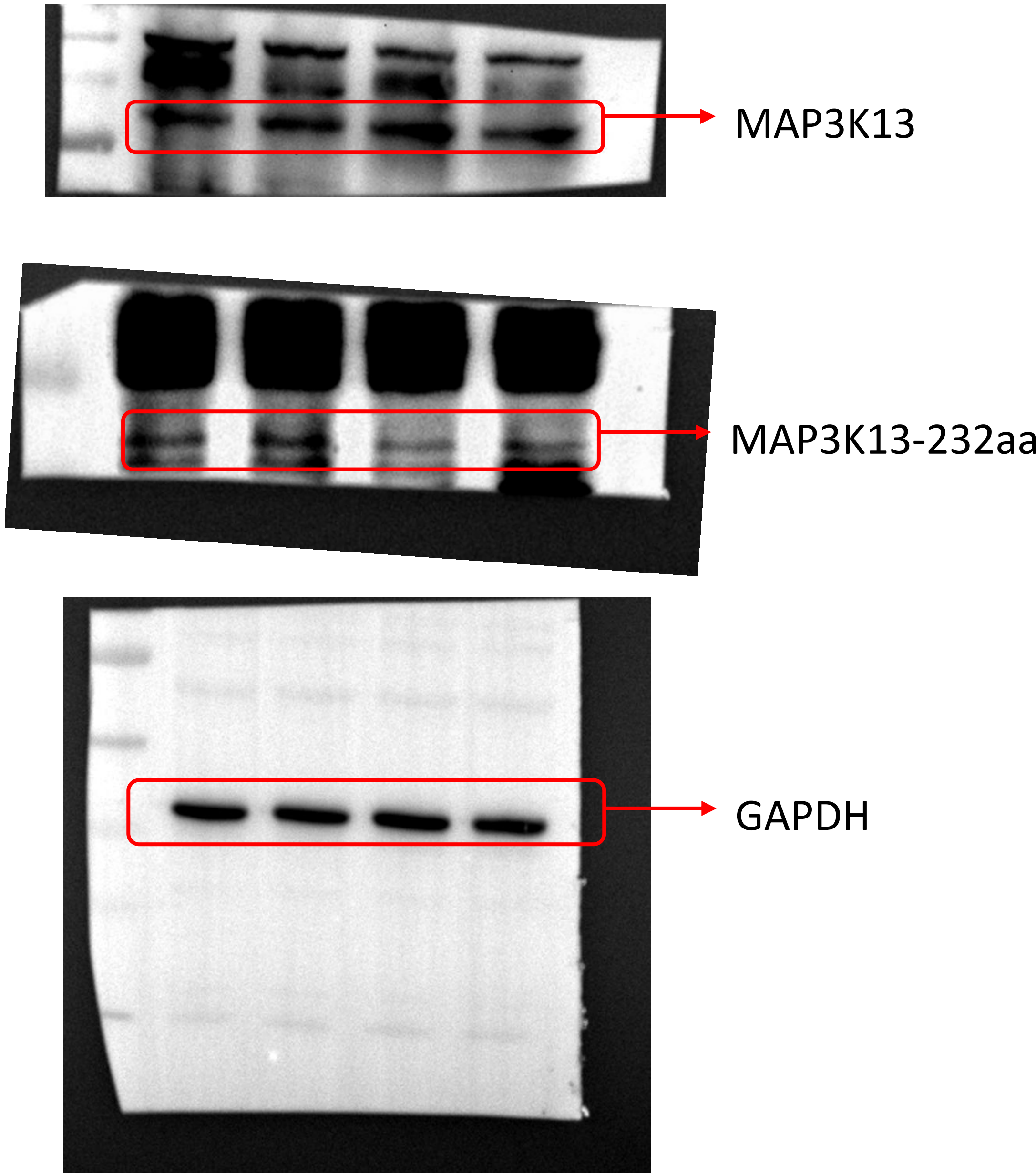

Fig.6D

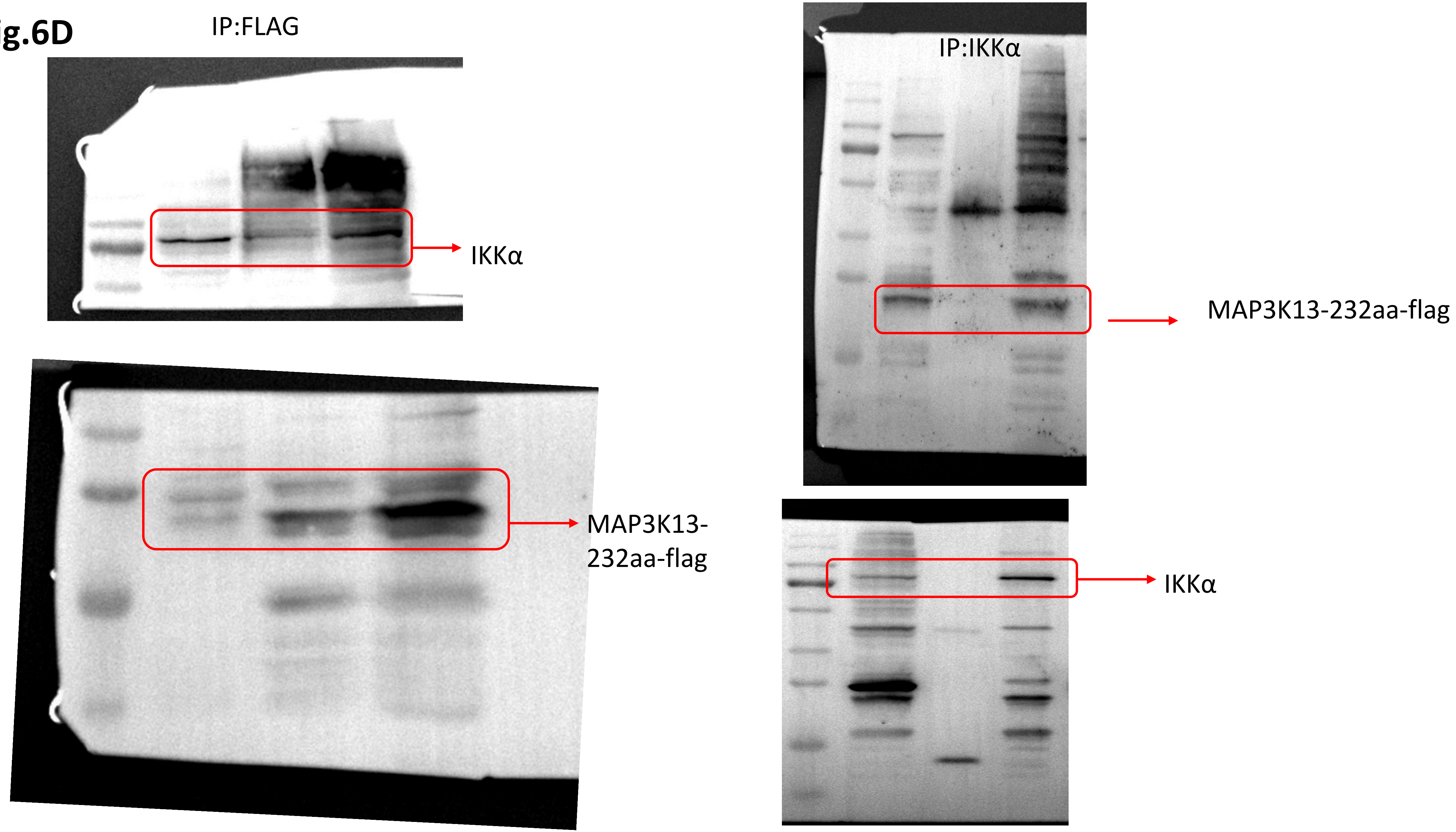
IP:FLAG
IKKα
MAP3K13-232aa-flag
IP:IKKα
MAP3K13-232aa-flag
IKKα

Fig.6F

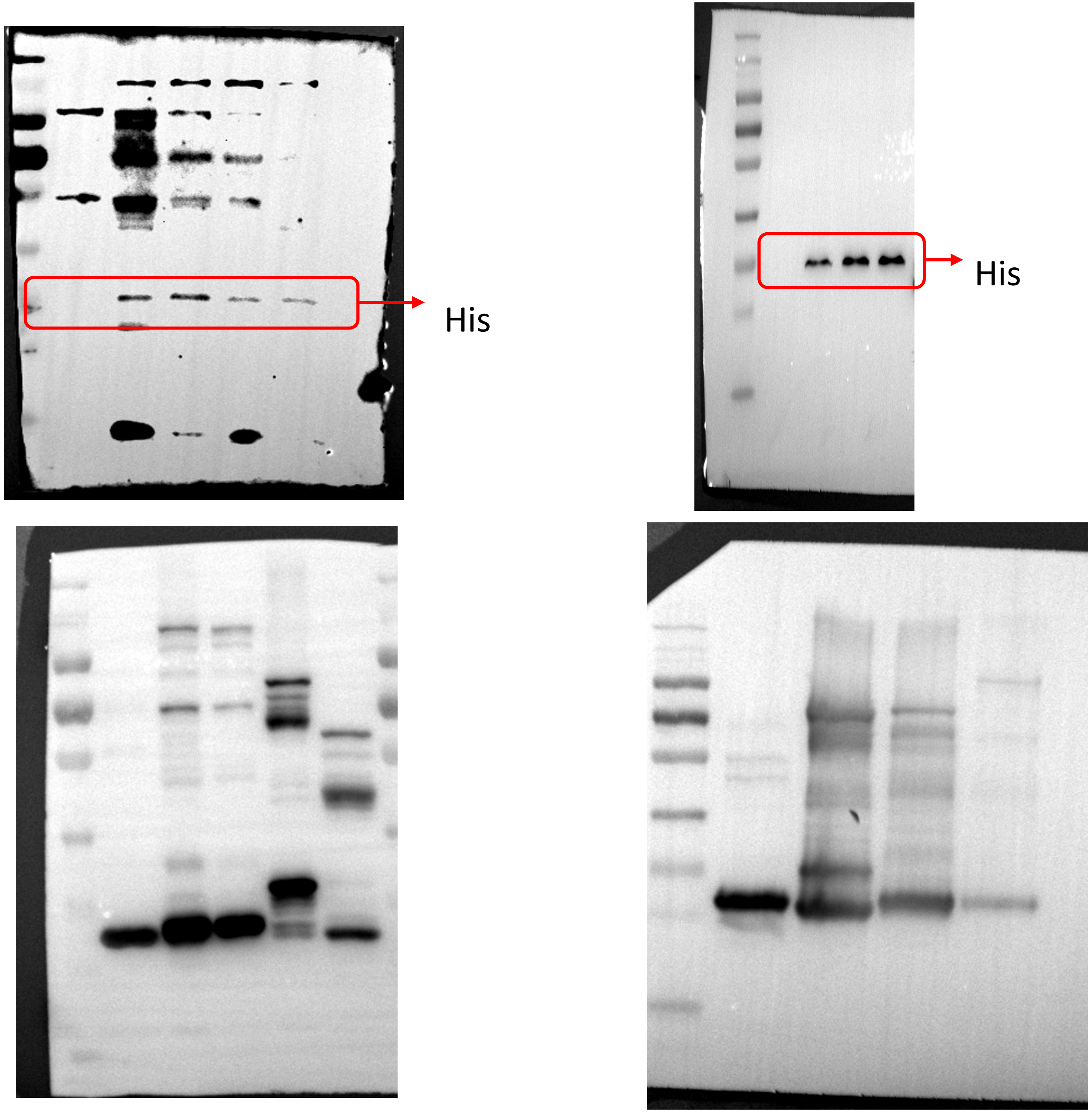

Fig.7A

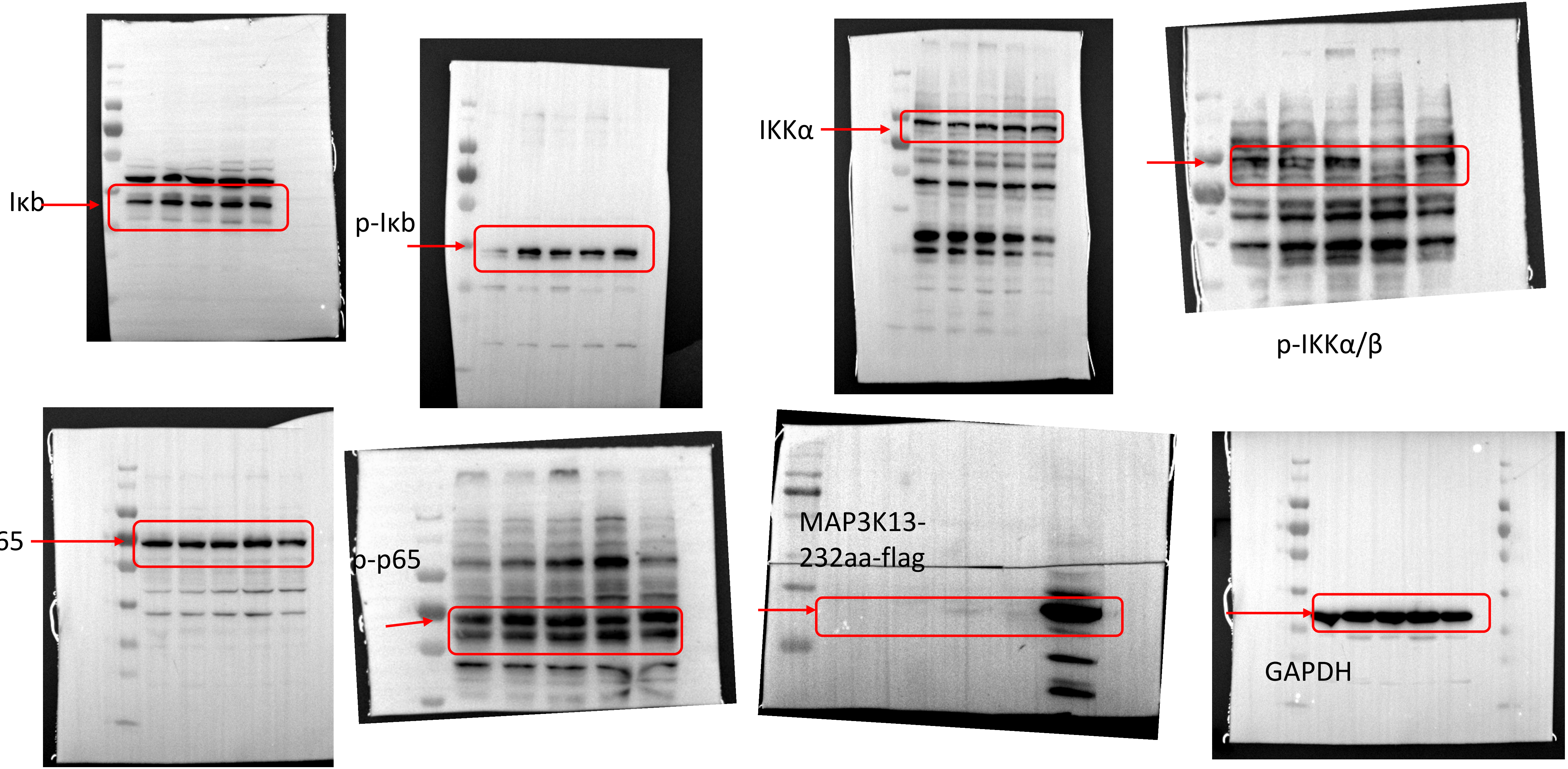

Fig.7B

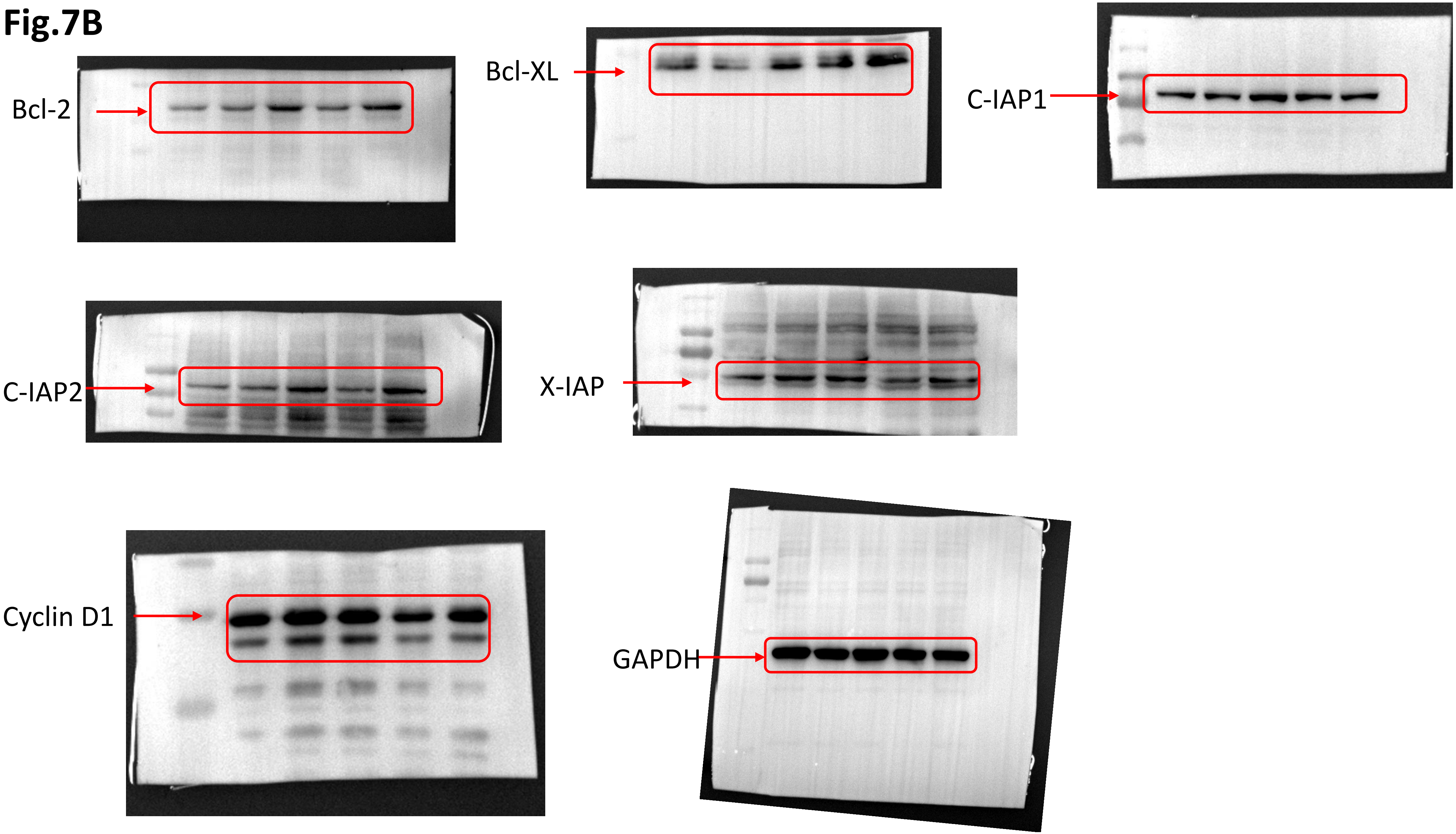

Fig.7F

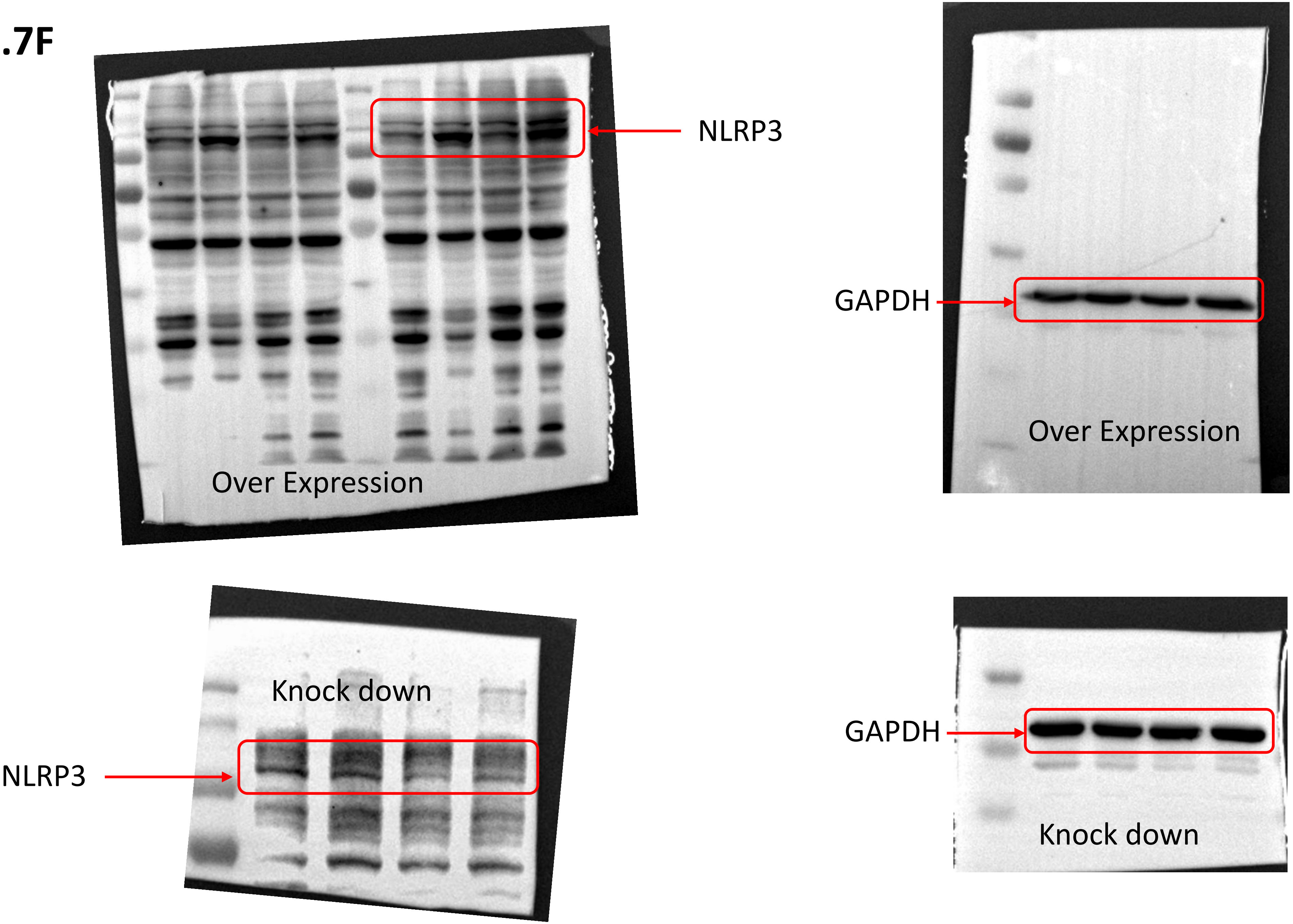
NLRP3
Over Expression
GAPDH
Over Expression
Knock down
NLRP3
GAPDH
Knock down

Fig.7G

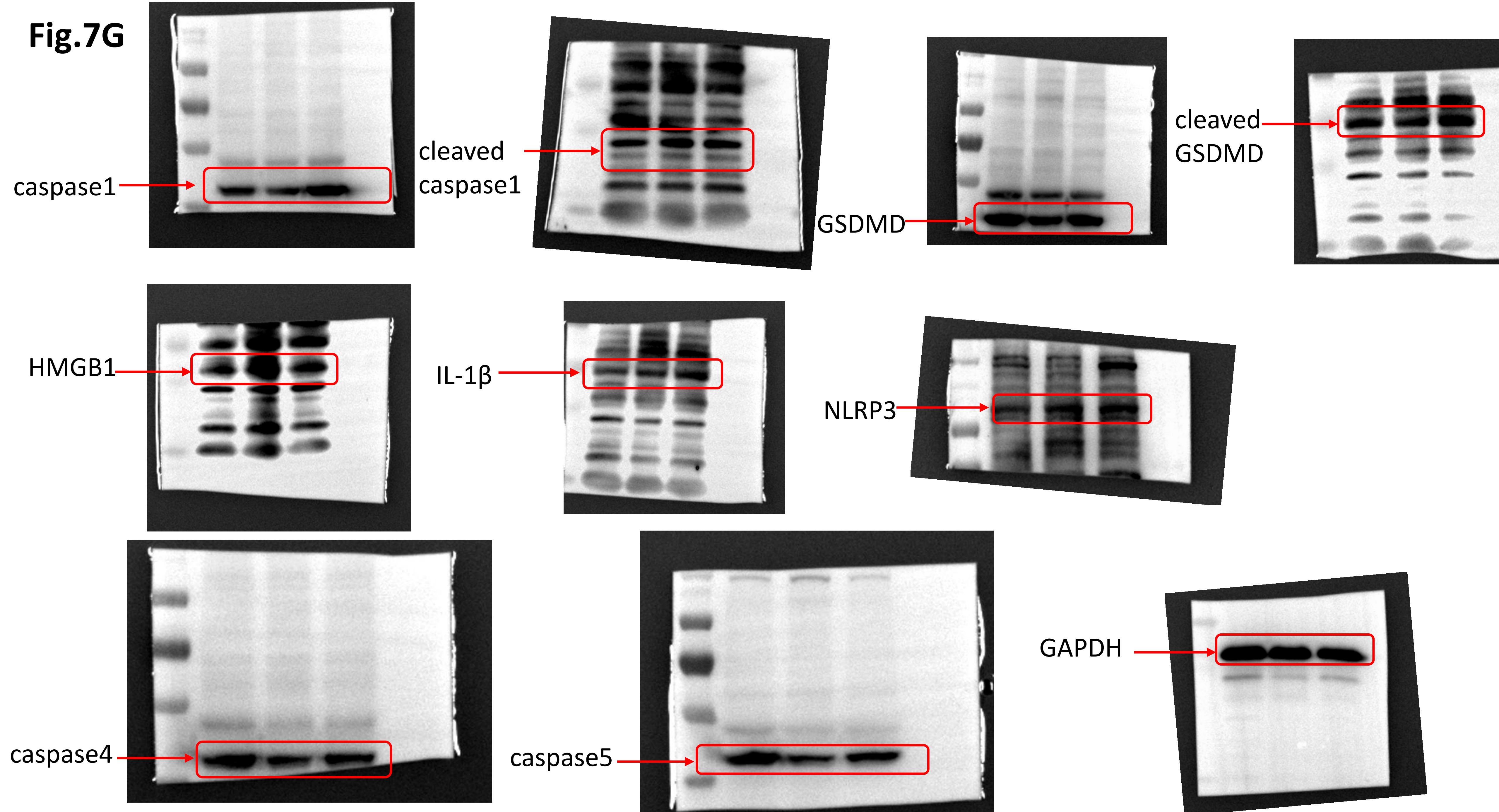
caspase1
cleaved caspase1
GSDMD
cleaved GSDMD
HMGB1
IL-1β
NLRP3
caspase4
caspase5
GAPDH

Fig.7H

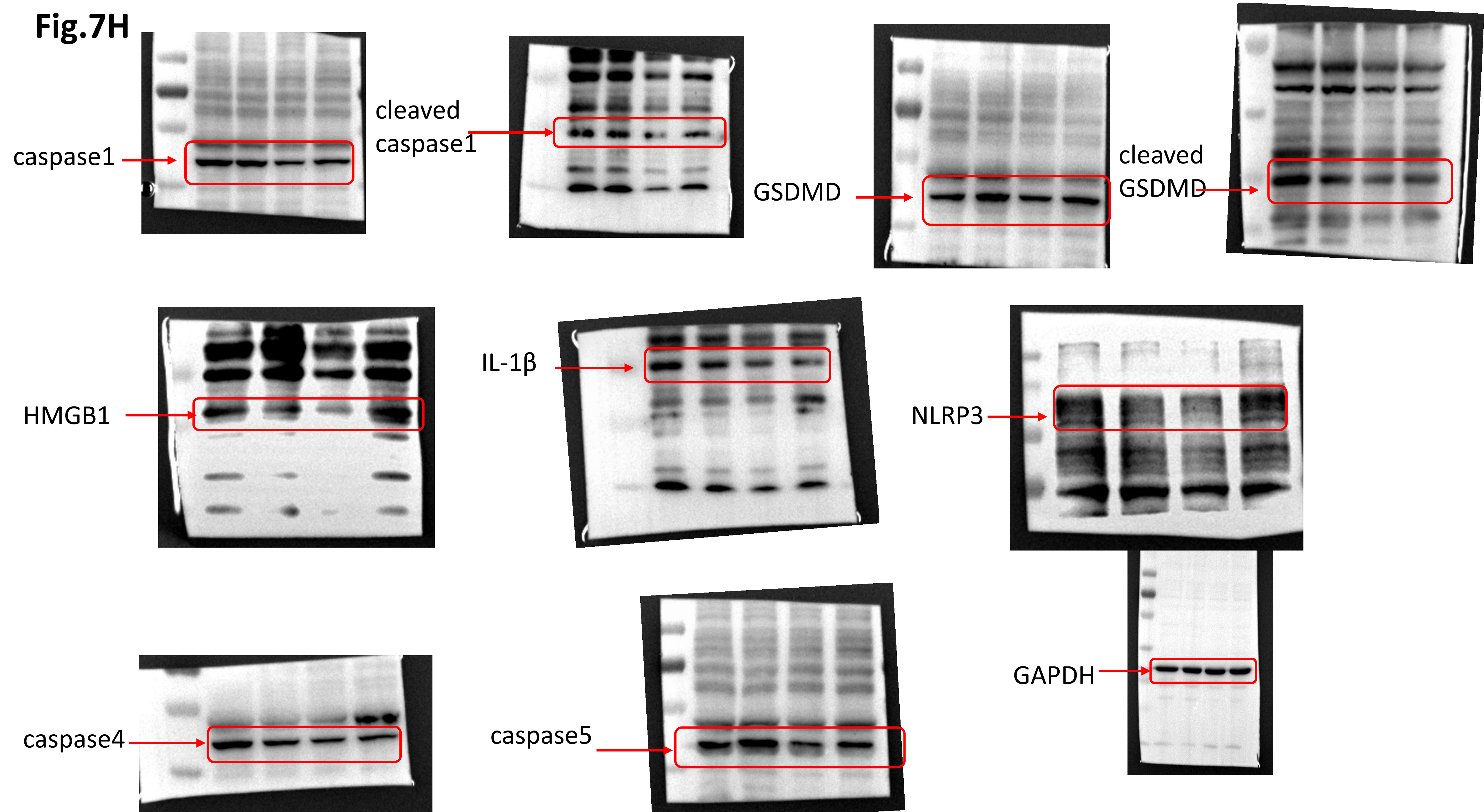
caspase1
cleaved caspase1
GSDMD
cleaved GSDMD
HMGB1
IL-1β
NLRP3
GAPDH
caspase4
caspase5

Fig.7I

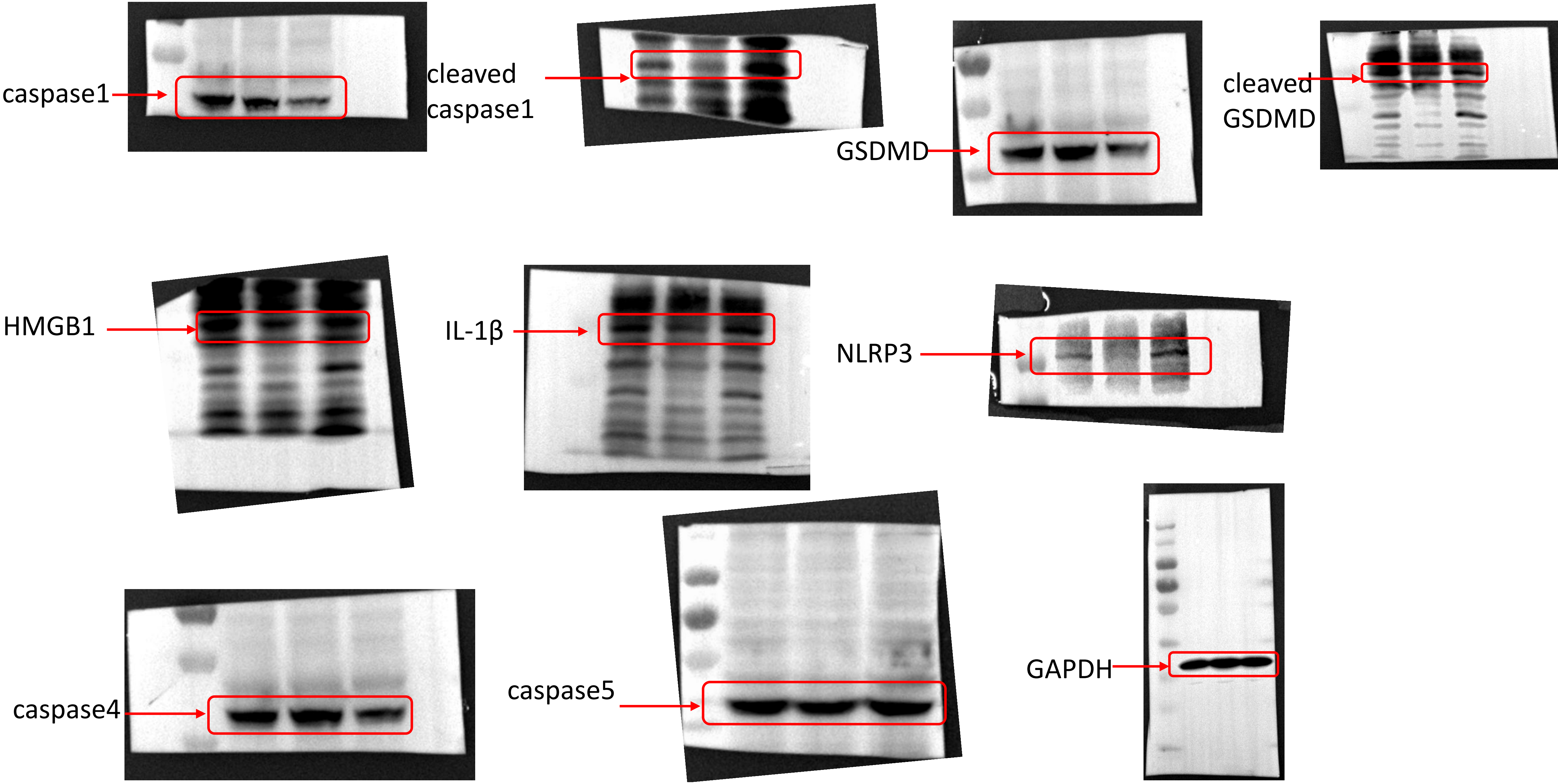

caspase1
cleaved caspase1
GSDMD
cleaved GSDMD
HMGB1
IL-1β
NLRP3
caspase4
caspase5
GAPDH

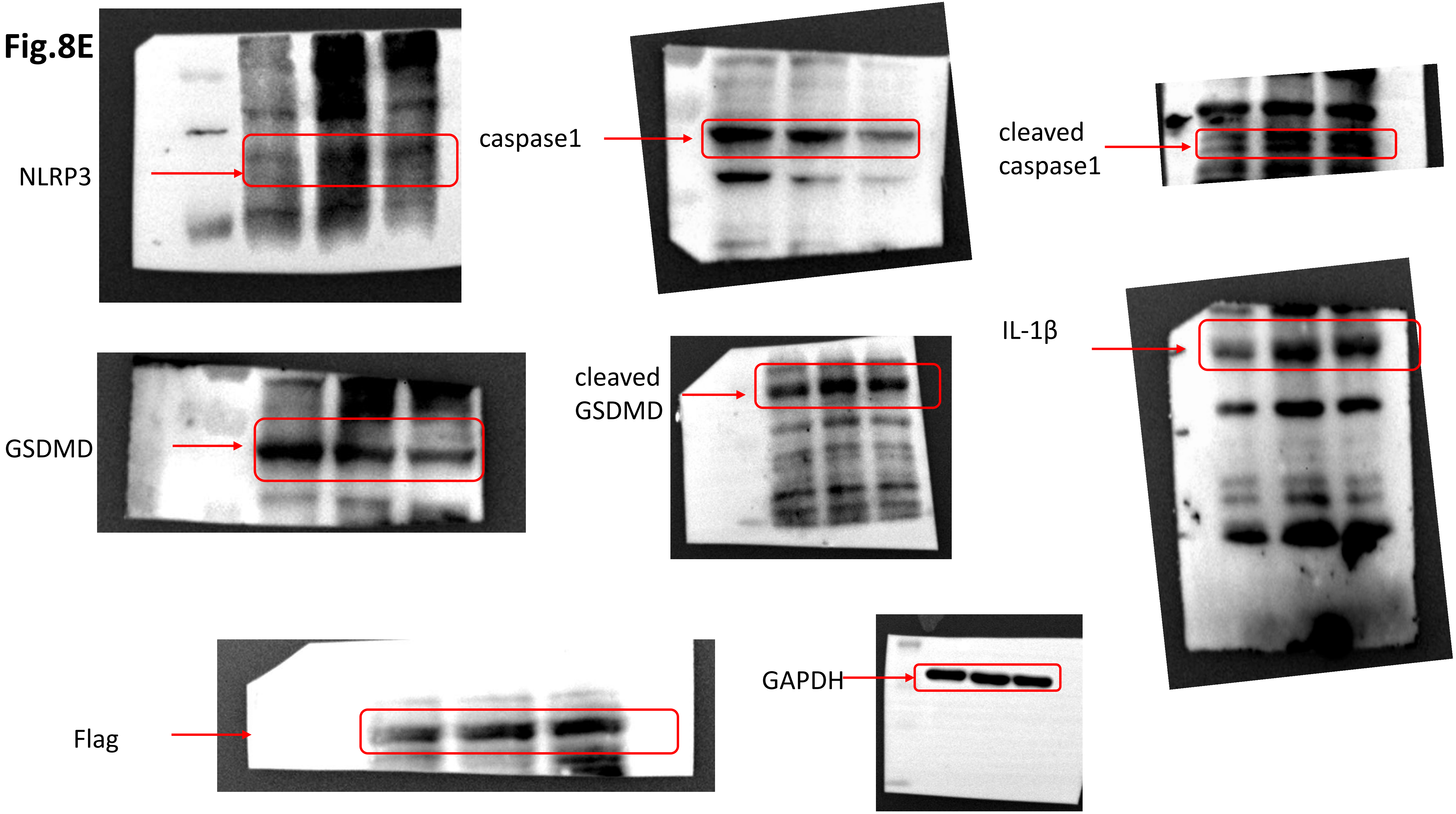
Fig.8E
NLRP3
caspase1
cleaved caspase1
GSDMD
cleaved GSDMD
IL-1β
Flag
GAPDH

S.2D

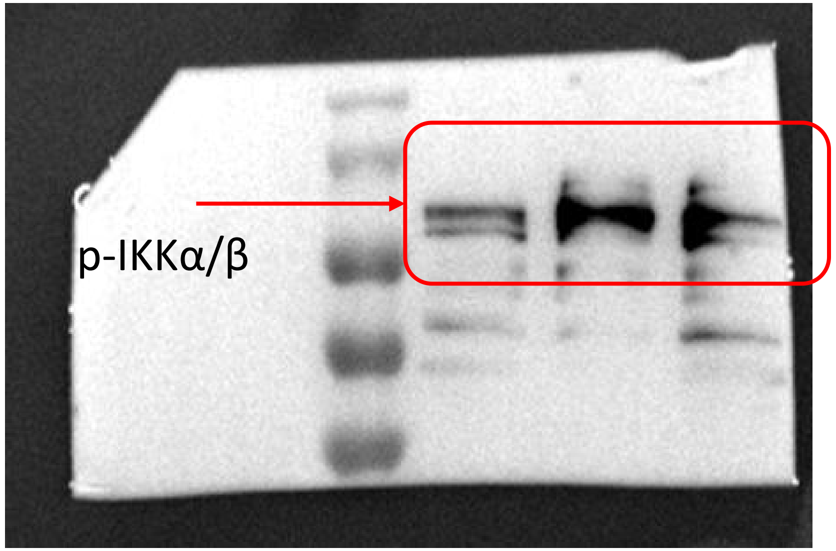

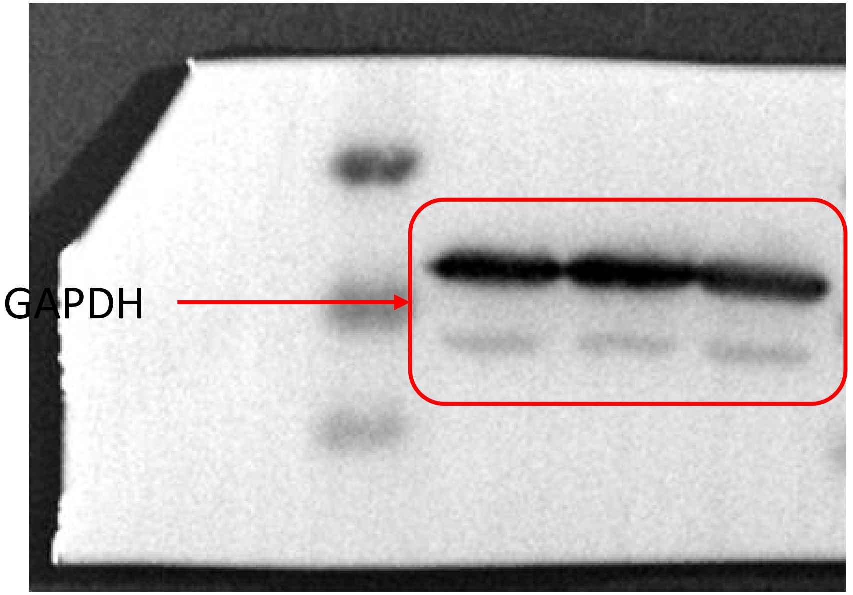

S.2E

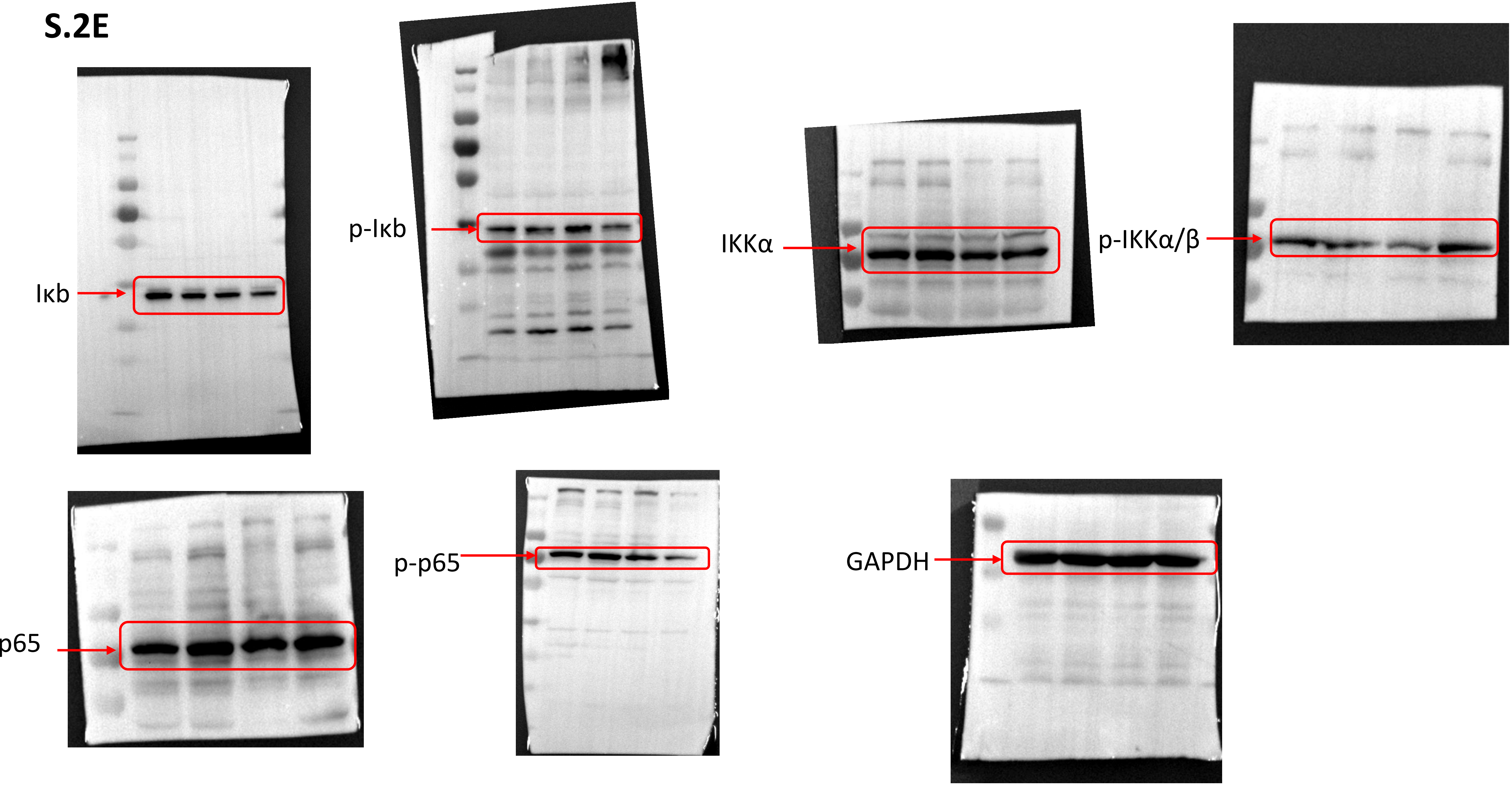

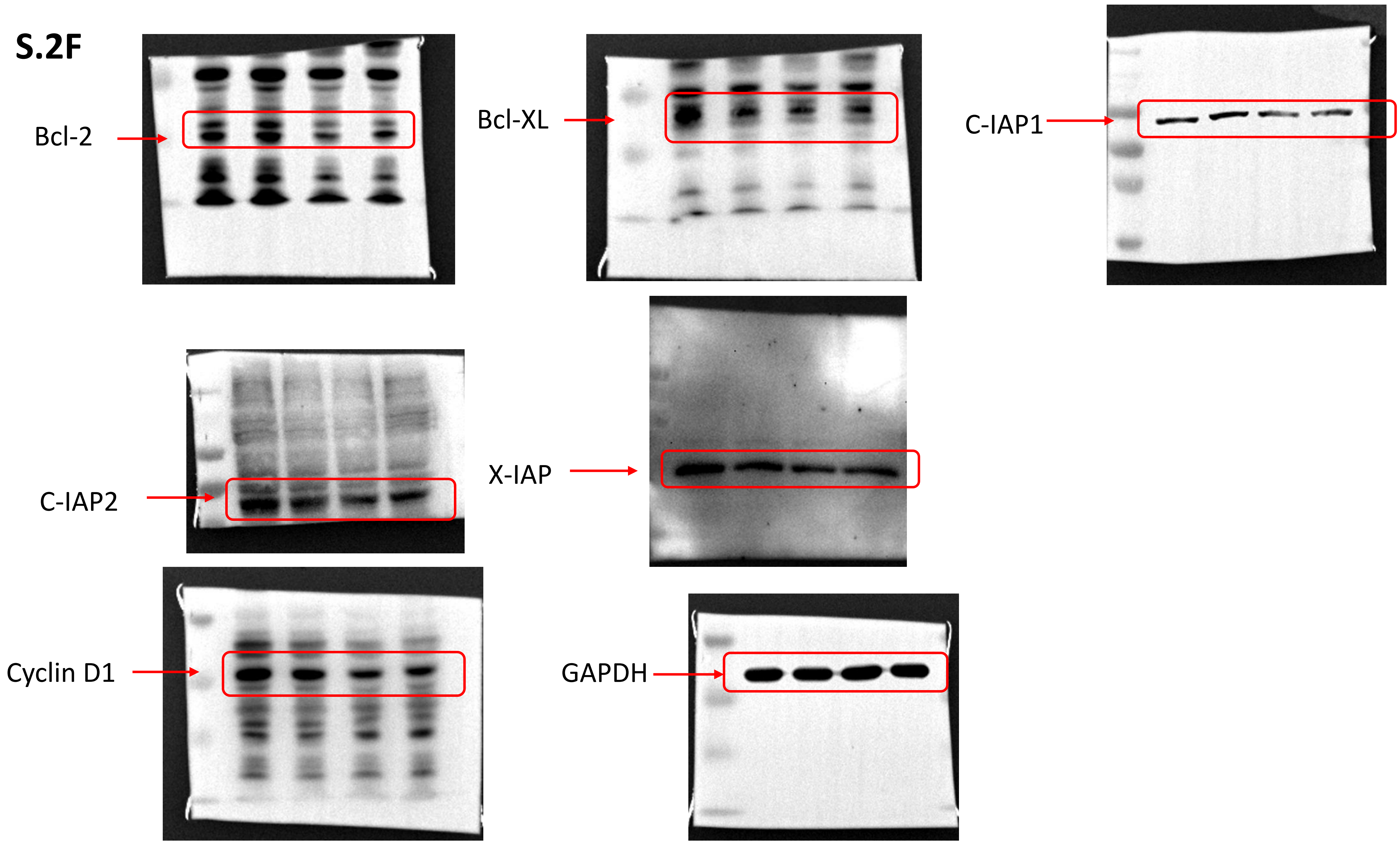
S.2F
Bcl-2
Bcl-XL
C-IAP1
C-IAP2
X-IAP
Cyclin D1
GAPDH

S.2G

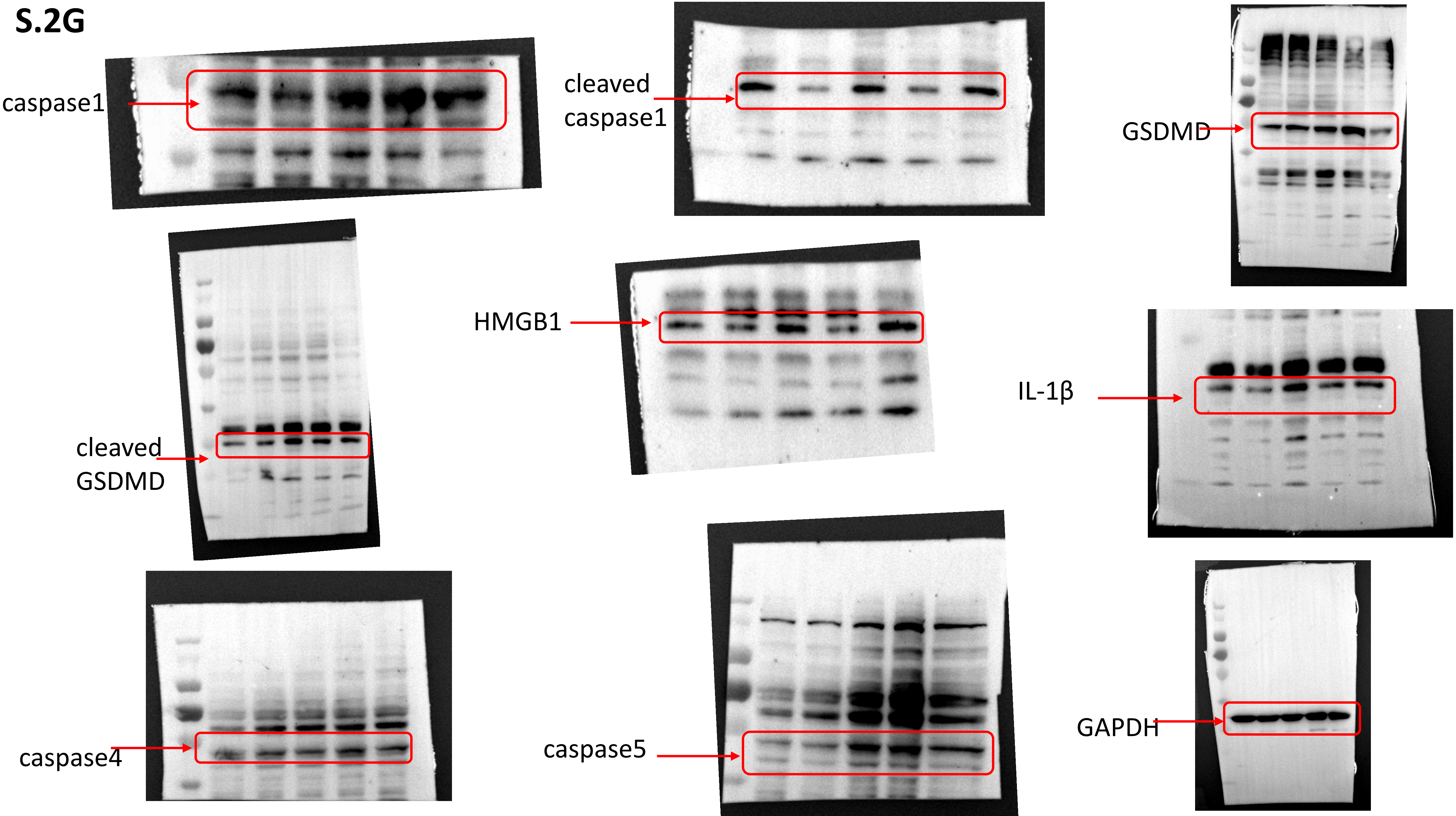
caspase1
cleaved caspase1
GSDMD
HMGB1
IL-1β
cleaved GSDMD
caspase4
caspase5
GAPDH

S.2H

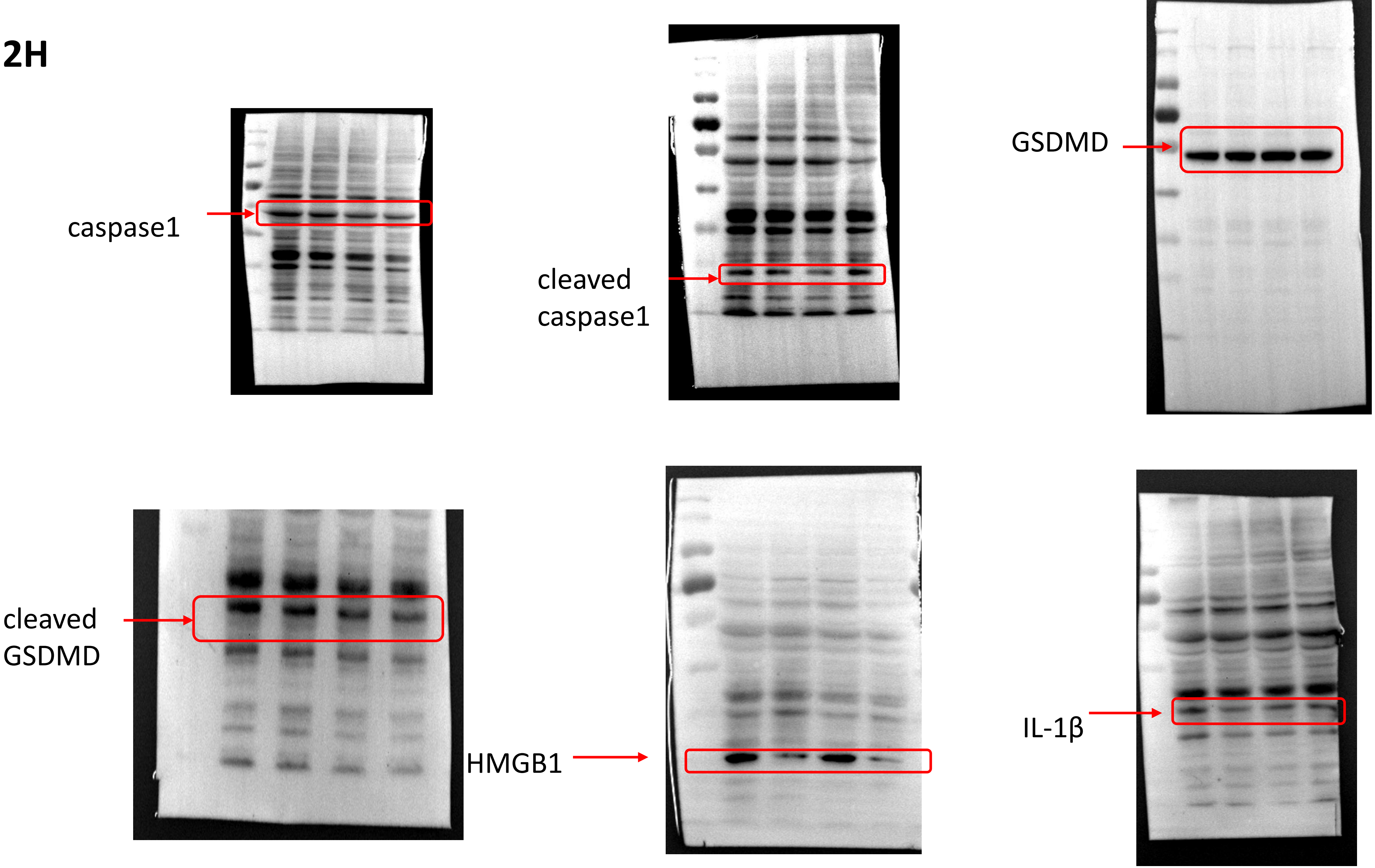

S.2H

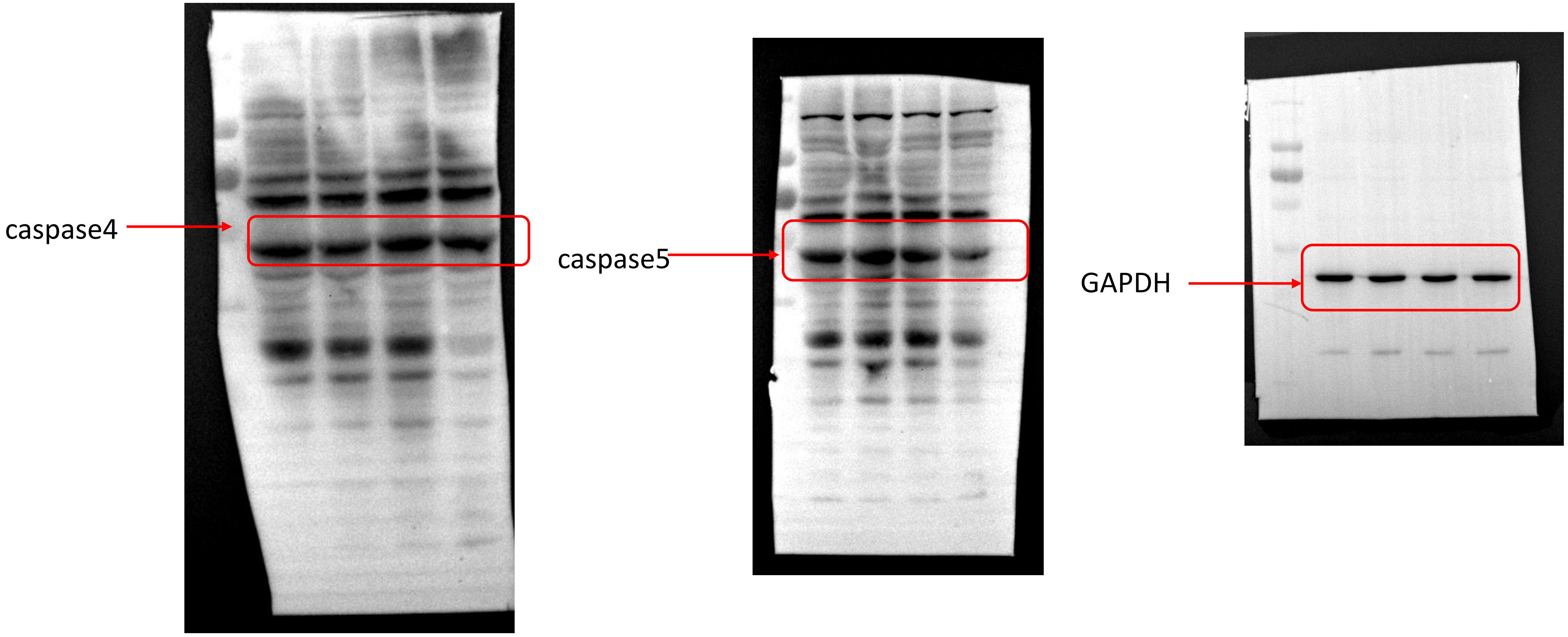

Supplement: Supplementary file 6 — western blot original blot [file 41419_2025_7991_MOESM6_ESM.pdf]
